# TRPC3-mediated NFATc1 calcium signaling promotes triple negative breast cancer migration through regulating glypican-6 and focal adhesion

**DOI:** 10.1007/s00424-024-03030-y

**Published:** 2024-10-22

**Authors:** Yan Wang, Xiaosheng Zhuang, Yanxiang Qi, Lung Yiu, Zhenping Li, Yuk Wah Chan, Xianji Liu, Suk Ying Tsang

**Affiliations:** 1 School of Life Sciences, The Chinese University of Hong Kong, Hong Kong SAR, China; 2 State Key Laboratory of Agrobiotechnology, The Chinese University of Hong Kong, Hong Kong SAR, China; 3 Key Laboratory for Regenerative Medicine, Ministry of Education, The Chinese University of Hong Kong, Hong Kong SAR, China; 4 Institute for Tissue Engineering and Regenerative Medicine, The Chinese University of Hong Kong, Hong Kong SAR, China

**Keywords:** TRPC3, NFATc1, Glypican 6, Focal adhesions, Triple-negative breast cancer, Cell migration

## Abstract

Canonical transient receptor potential isoform 3 (TRPC3), a calcium-permeable non-selective cation channel, has been reported to be upregulated in breast cancers and a modulator of cell migration. Calcium-sensitive transcription factor NFATc1, which is important for cell migration, was shown to be frequently activated in triple negative breast cancer (TNBC) biopsy tissues. However, whether TRPC3-mediated calcium influx would activate NFATc1 and affect the migration of TNBC cells, and, if yes, the underlying mechanisms involved, remain to be investigated. By immunostaining followed by confocal microscopy, TNBC lines MDA-MB-231 and BT-549 were both found to express TRPC3 on their plasma membrane while ER^+^ line MCF-7 and HER2^+^ line SK-BR3 do not. Blockade of TRPC3 by pharmacological inhibitor Pyr3 or stable knockdown of TRPC3 by lentiviral vector both inhibited cell migration as measured by wound healing assay. Importantly, blocking TRPC3 by Pyr3 or knockdown of TRPC3 both caused the translocation of NFATc1 from the nucleus to the cytosol as revealed by confocal microscopy. Interestingly, NFATc1 was found to bind to the promoter of glypican 6 (GPC6) as determined by chromatin immunoprecipitation assay. Consistently, knockdown of TRPC3 decreased the expression of GPC6 as revealed by western blotting. Moreover, long-term knockdown of GPC6 by lentiviral vector also consistently decreased the migration of TNBC cells. Intriguingly, GPC6 proteins physically interact with vinculin in MDA-MB-231 as determined by co-immunoprecipitation. Blockade of TRPC3, knockdown of TRPC3 or knockdown of GPC6 all induced larger, stabilized actin-bound peripheral focal adhesion (FA) formations in TNBC cells as determined by co-staining of actin and vinculin followed by confocal microscopy. These large, stabilized actin-bound peripheral FAs indicated a defective FA turnover, and were reported to be responsible for impairing directed cell migration. Our results suggest that, in TNBC cells, calcium influx through TRPC3 channel positively regulates NFATc1 nuclear translocation and GPC6 expression, which maintains the dynamics of FA turnover and optimal cell migration. Our study reveals a novel TRPC3-NFATc1-GPC6-vinculin signaling cascade in maintaining the migration of TNBC cells.

**Supplementary Information:**

The online version contains supplementary material available at 10.1007/s00424-024-03030-y.

## Background

Triple-negative breast cancer (TNBC) is a highly aggressive and invasive breast cancer subtype with poor prognosis [20]. According to the statistics from the National Cancer Institute (SEER 18, 2010–2016), the 5-year relative survival rate of female patients with metastatic breast cancer is only about 28.1%. Metastatic breast cancer is lethal usually because the breast tumor cells spread and colonize at distant vital organs, and make the vital organ dysfunction. Inhibition of metastatic TNBC cell migration is still challenging due to the lack of effective molecular target [11].

Calcium is a vital signaling cation involved in regulating numerous cellular processes. Intracellular calcium level is regulated by the interplay of a number of ion channels, exchangers and pumps located both in the plasma membrane and on intracellular organelles. For ion channels in the plasma membrane, transient receptor potential (TRP) channel, a group of non-selective cation channels, is one of the major players [24]. Calcium entry through TRP channels has been reported to promote metastatic cancer cell migration [16]. On the other hand, disruptions of intracellular calcium signaling transductions, changes in focal adhesion (FA) dynamics and cytoskeletal organizations in response to TRP channel suppression were observed in cancer cells [8, 47].

Calcium-sensitive nuclear factor of activated T cells (NFAT) proteins were found to be over-expressed or activated in tumor cells including breast, colon, pancreatic and aggressive immune cells [28]. NFATc1 was shown to be frequently activated in TNBC as evaluated in tumor tissues from patients, and nuclear localized NFATc1 in TNBC cells was reported to be important for cell migration [34].

Canonical transient receptor potential isoform 3 (TRPC3), a calcium-permeable non-selective cation channel, has been reported to be upregulated in breast cancer biopsy tissues [2] and a modulator of cancer cell migration [29]. In the present study, we aimed to investigate if the long term knockdown of TRPC3 would affect the migration of TNBCs, and, if yes, the underlying mechanisms involved. The novel information generated in the present study shall shed light on future therapeutic targets for TNBC treatment at molecular level.

## Methods

### Cell culture

Four human breast cancer cell lines, MDA-MB-231 (ER-, PR- and HER2-, ATCC HTB-26), BT-549 (ER-, PR- and HER2-, ATCC HTB-122), MCF-7 (ER^+^, PR^±^ and HER^2−^, ATCC HTB-22), and SK-BR3 (ER^−^, PR^−^, and HER^2+^, ATCC HTB-30) (American Type Culture Collection, Manassas, VA, USA) were selected as our in vitro research models. MDA-MB-231, MCF-7, SK-BR3 and BT-549 were cultured at 37 °C under an atmosphere of 5% CO_2_/95% air. MDA-MB-231 and MCF-7 cells were cultured in the phenol red-free RPMI 1640 medium (Invitrogen, Carlsbad, CA, USA) supplemented with 10% heat-inactivated fetal bovine serum (FBS) (Invitrogen). The complete medium for BT-549 is RPMI-1640 (ATCC modification) (Invitrogen) supplemented with 0.023U/ml insulin (Thermo Fisher Scientific, Waltham, MA, USA) and 10% FBS, whereas the complete medium for SK-BR3 is McCoy’s 5A (Modified) Medium (Invitrogen) supplemented with 10% FBS.

### Lentiviral vector production and generation of stable cell lines

The lentiviral vector pLKO.1 harboring short hairpin RNA (shRNA) targeting TRPC3 (shTRPC3) was purchased from Dharmacon (part of GE Healthcare, Lafayette, CO, USA); lentiviral vector pLKO.1 harboring shRNA targeting GPC6 (shGPC6) was purchased from Sigma-Aldrich (Sigma-Aldrich, Missouri, MO, USA); lentiviral vector pLKO.1 harboring non-targeting control shRNA (shCtrl) was made as previously described [10, 43]. shRNA targeting sequences were listed as follows:

- pLKO.1-shTRPC3: TRCN0000044031, 5’-CCCACCAGAGATACAGTATTT-3’;
- pLKO.1-shGPC6: TRCN0000123097, 5’-GCACAGCAAAGCCAGATACTT-3’;
- pLKO.1-shCtrl: 5’-CCTAAGGTTAAGTCGCCCTCG-3’.

Helper plasmids psPAX2 and pMD2.G were gifts from Professor Didier Trono (Addgene plasmid # 12,260; http://n2t.net/addgene:12260; RRID:Addgene_12260; Addgene plasmid # 12,259; http://n2t.net/addgene:12259; RRID:Addgene_12259). Lentiviral particles were generated by HEK293T cells. 5.0 μg lentiviral vectors and helper plasmids 10 μg psPAX2 and 2.5 μg pMD.G were co-transfected into HEK293T cells (90% confluency of 100-mm dishes) using 50 μL lipofectamine (Invitrogen, Carlsbad, CA, USA) in 18 mL Opti-MEM (Invitrogen) per 100-mm dish. 6 h later, Opti-MEM medium was replaced by normal HEK293T culture medium [DMEM (Invitrogen) supplemented with 10% FBS, 2 mM L-glutamine, 0.1 mM NEAA, 50 U/mL penicillin, 50 μg/mL streptomycin]. The supernatants containing lentiviruses were harvested at 48 h post-transfection and filtered through 0.45-μm pore-size filters (Millipore, Bedford, MA) and stored at -80 °C before use. MDA-MB-231 cells were infected by lentiviruses [(virus supernatant: MDA-MB-231 medium = 1: 4 (v:v)] for 48 h and then selected by puromycin (1.5 μg/mL) for 10 days to generate stable cell lines MDA-MB-231-shCtrl, MDA-MB-231-shTRPC3 and MDA-MB-231-shGPC6 for further experiments. These cells were cultured in RPMI 1640 medium (Invitrogen) supplemented with 5% heat-inactivated FBS (Invitrogen), at 37 °C under an atmosphere of 5% CO_2_/ 95% air.

### Western blot

MDA-MB-231, MDA-MB-231-shCtrl, MDA-MB-231-shTRPC3 and MDA-MB-231-shGPC6 cell lysates were prepared and Western blot was performed as previously described [22]. To assay for the presence of TRPC3, 1:1000 rabbit anti-TRPC3 (Alomone, Jerusalem, Israel) and 1:1000 mouse anti-TRPC3 (Santa Cruz, Dallas, TX, USA) were used. To assay for the expression of GPC6, 1:1000 mouse anti-GPC6 (Invitrogen) was used. In all cases, the membranes were stripped and probed with 1:1000 rabbit anti-β-tubulin (Cell Signaling, Danvers, MA, USA) as an internal control. After primary antibody probing, membranes were washed in TBST, and incubated with HRP-conjugated secondary antibody (Dako, Glostrup, Denmark) in the dilution of 1:3000 for 1 h at room temperature. Protein expression was detected by enhanced chemiluminescent substrate (Pierce, Thermo Fisher Scientific, Waltham, MA, USA) and protein bands were visualized by film exposure. The density of the bands was quantified using Image J software (version 1.48v, National Institutes of Health, Bethesda, MD, USA).

### Immunocytochemistry and fluorescence imaging

Immunocytochemistry was performed as previously described [22, 46]. Cells were fixed with 2% paraformaldehyde (Sigma-Aldrich) for 10 min at 37 °C, then rinsed in PBS twice for 5 min, and subsequently incubated in 0.1% Triton X-100 (Sigma-Aldrich) for 15 min. Coverslips were then washed with PBS twice, and incubated in a blocking solution containing 2% BSA and 5% normal goat serum (NGS) (Invitrogen) for 1 h followed by an overnight incubation in the blocking solution containing primary antibodies at 4 °C in the dark. To assay for the presence of TRPC3, cells on coverslips were incubated with 1:100 rabbit anti-TRPC3 (Abcam, Cambridge, UK) and 1:100 mouse anti-TRPC3 (Abnova, Taipei, Taiwan), respectively. To assay for the expression of vinculin, 1:1000 mouse anti-vinculin (Abcam) was used. To assay for the presence of NFATc1, 1:100 mouse anti-NFATc1 (Invitrogen) was used. After three times being washed with PBS supplemented with 0.1% Tween (Sigma-Aldrich), secondary antibodies, 1:100 Alexa Fluor 488 goat anti-mouse (Invitrogen) or 1:100 Alexa Fluor 488 goat anti-rabbit (Invitrogen) (both diluted in 1% NGS/PBS), were applied to incubate the cover slides for 1 h at room temperature. Then 1:5000 DAPI (Roche, Basel, Switzerland) in PBS was used to stain nuclei for 10 min at room temperature, while 1:40 phalloidin 568 (Thermo Fisher Scientific) was used to stain actin for 20 min at room temperature. Slides were affixed with mounting medium (Dako, Carpinteria, CA, USA) and viewed using an Olympus FluoView FV1000 confocal laser scanning microscope with a 60 × objective. Images were analyzed using the FV1000 software (Olympus, Tokyo, Japan). The area and length of actin-binding vinculin and the nuclear-to-cytoplasmic fluorescence ratios of the NFAT signals were measured using Image J software. The NFAT fluorescence data were analyzed as the fluorescence signal multiplied with the cell/nucleus area.

### Confocal Ca^2+^ imaging

Confocal Ca^2+^ imaging using Fluo-4 AM (Thermo Fisher Scientific) was performed as previously described [33]. Drugs including adenosine-triphosphate disodium salt hydrate (ATP) (Sigma) and CaCl_2_ (Sigma) were added at specified concentrations at a given time. Raw traces reflecting the changes in cytosolic Ca^2+^ level were expressed as F/F0 which was defined by the fluorescence intensity at a given time normalized to its baseline. Data was analyzed using with FV1000 software (Olympus).

### Wound healing assay

4 × 10^5^ of MDA-MB-231-shCtrl, MDA-MB-231-shTRPC3 and MDA-MB-231-shGPC6 cells were seeded in a 12-well plate for 24 h in normal culture medium. After 24 h, cells normally reached 95% confluency. The cells were incubated with 5 μg/mL mitomycin C (Sigma) for 2 h prior to the scratch assay.

In another experiment, 1.8 × 10^5^ BT-549 cells were seeded in a 24-well cell culture plate (Cat no. 662 160, Greiner Bio-One, Kremsmünster, Austria) in the normal cell culture medium (pH = 7.4). After 24 h, the cells normally reached 95% confluency. The cells were incubated with 5 μg/mL mitomycin C (Sigma) for 2 h, after which the solution was replaced with normal medium supplemented with DMSO, 3 μM or 10 μM Pyr3 prior to the scratch assay.

Then, p1000 blue tips were applied for wound scratch and fresh medium was replaced. Images were captured at indicated hours (0, 16, or 24 h) after scratch using Nikon TE300 eclipse microscope with a 4 × objective. Ratio of the wound area to the whole area in the picture was measured at each time point. Cell migration rate was calculated as (Wound area at 0 h-Wound area at 24 h)/ Wound area at 0 h × 100%.

### NFATc1 DNA plasmid transfection and confocal imaging

EGFPC1-huNFATc1EE-WT was a gift from Jerry Crabtree (Addgene plasmid # 24,219; http://n2t.net/addgene:24219; RRID:Addgene_24219) (Addgene, Cambridge, MA, USA) [3]. 24 h before transfection, MDA-MB-231-shCtrl and MDA-MB-231-shTRPC3 cells were seeded at 4 × 10^4^ cells/well to gelatin-coated 24-well plate dishes. For each transfection sample, 1.0 μg plasmid was diluted in 50 μL Opti-MEM (Invitrogen), and mixed with 2 μL Lipofectamine 2000 (Invitrogen) in 50 μL Opti-MEM for transfection of cells in 400 μL fresh medium without antibiotics. Cells were incubated at 37 °C with 5% CO_2_, medium was changed after 6 h and testing for transgene expression was performed at 48 h post-transfection. Cells with EGFP-NFATc1 protein expressed were observed as described above under ‘Fluorescence imaging’. For data analysis, areas of nuclei and cytoplasm of various cells were randomly chosen from the captured images; fluorescence intensities of the corresponding areas were used to calculate the nuclear-to-cytoplasmic signal ratio of each cell.

### Co-immunoprecipitation (Co-IP) assay

MDA-MB-231 cells were seeded in 100-mm dishes (three dishes of cells in 90% confluency were used for one group). Cell pellets were collected and lysed using Co-IP lysis buffer (20 mM Tris, pH 7.4, 150 mM NaCl, 5 mM MgCl_2_, 0.5% NP-40) containing protease inhibitor cocktails. Cell lysates were used in immunoprecipitation after centrifuged at 12,000 rpm for 20 min. Proteins extracted from the cells were pre-incubated with primary antibodies, 1:1000 mouse anti-vinculin (Abcam)/ 1:1000 mouse anti-GPC6 (Invitrogen)/ 1:1000 normal mouse control IgG (Santa Cruz), at 4 °C for 3 h, and then incubated with 20 μL Protein A/G agarose beads (Santa Cruz) for overnight with gentle rotation. The immunocomplexes were precipitated by centrifugation at 3,000 rpm for 5 min, and the precipitates were washed three times with washing buffer. The precipitates were resuspended in 6 × SDS-PAGE sample loading buffer and boiled (100 °C) for 10 min. Then, the samples were loaded into SDS-PAGE for Western blotting.

### Chromatin immunoprecipitation (ChIP)—Quantitative Polymerase Chain Reaction (qPCR) (ChIP-qPCR) assay

Chromatin immunoprecipitation (ChIP) assay was performed following the manual of Millipore ChIP Assay Kit (17–295, EMD Millipore Co., Temecula, CA). Cells were cross-linked with 1% formaldehyde at 37 °C for 10 min in ice-cold PBS containing protease and phosphatase inhibitors. Chromatin was isolated from pelleted nuclei by 1% SDS lysis buffer [which included 1% SDS, 10 mM EDTA, and 50 mM Tris (pH 8.1)] (20–163, EMD Millipore Co.) and sheared by gentle sonication in ice-water bath using a Bransonic sonicator (Branson cleaning equipment Co., Shelton, CN, USA) to 200–500 bp fragments. Soluble chromatin was precleared with protein A/G agarose (Millipore) for 30 min and immunoprecipitated with antibodies against ChIP- grade IgG or NFATc1 (Abcam) overnight at 4 °C. Cross-link was reversed with 0.2 M NaCl for 4 h at 65 °C. DNA was extracted as previously described [45] and was purified using the MaXtract high density columns (129056, Qiagen, Merck KGaA, Darmstadt, Germany). Purified DNA was used as a template for real-time quantitative PCR (qPCR) amplification followed by agarose gel electrophoresis (2%). 10% of inputs was set as control. Transcription factor-binding sites were analyzed in GPC6 promoter sequence downloaded from the Eukaryotic Promoter Database (EPD) (https://epd.epfl.ch/search_EPDnew.php?query=GPC6&db=human).

The primers used for the GPC6 promoter were as follows:

- GPC6-1 (-1511 to -1407), 5’-CAACCTCACCGTTTGTCCAC-3’ and 5’-TTTATGCTAACTGGCAGATC-3’;
- GPC6-2 (-1285 to -1147), 5’-GGTTTTGTTTTGTCTTGACC-3’ and 5’-TTCCCCCTCTCCTAACCACC-3’;
- GPC6-3 (-1078 to -979), 5’-CTTCATGAGTCATTCTGGTC-3’ and 5’-TACTACAAGGTAATGCACAG-3, these three primer pairs include both the proximal NFATc1 and the AP-1 binding sites;
- GPC6-4 (-871 to -754), 5’-GGAGCTCTGGGTTTCAACAC-3’ and 5’-CACTGATGTTCTGTGAACGG-3, which includes only the proximal AP-1 binding site;
- GPC6-5 (-551 to -410), 5’-CCCGATCCTCCACACATTAG-3’ and 5’-GGCACACATTACAGACTGGT-3, which includes only the proximal NFATc1 binding site.

### Statistical analysis

Each experiment was repeated at least three times. The results were expressed as mean ± SEM. Statistical significance between two groups of means was determined by student’s t-test. Statistical significance between three or more groups of means was determined by analysis of variance (ANOVA) with post-hoc Turkey test. *P* < 0.05 was considered to be statistically significant.

## Results

### TPRC3 blockade or long-term knockdown of TRPC3 both inhibited TNBC migration

**Fig. 1:**
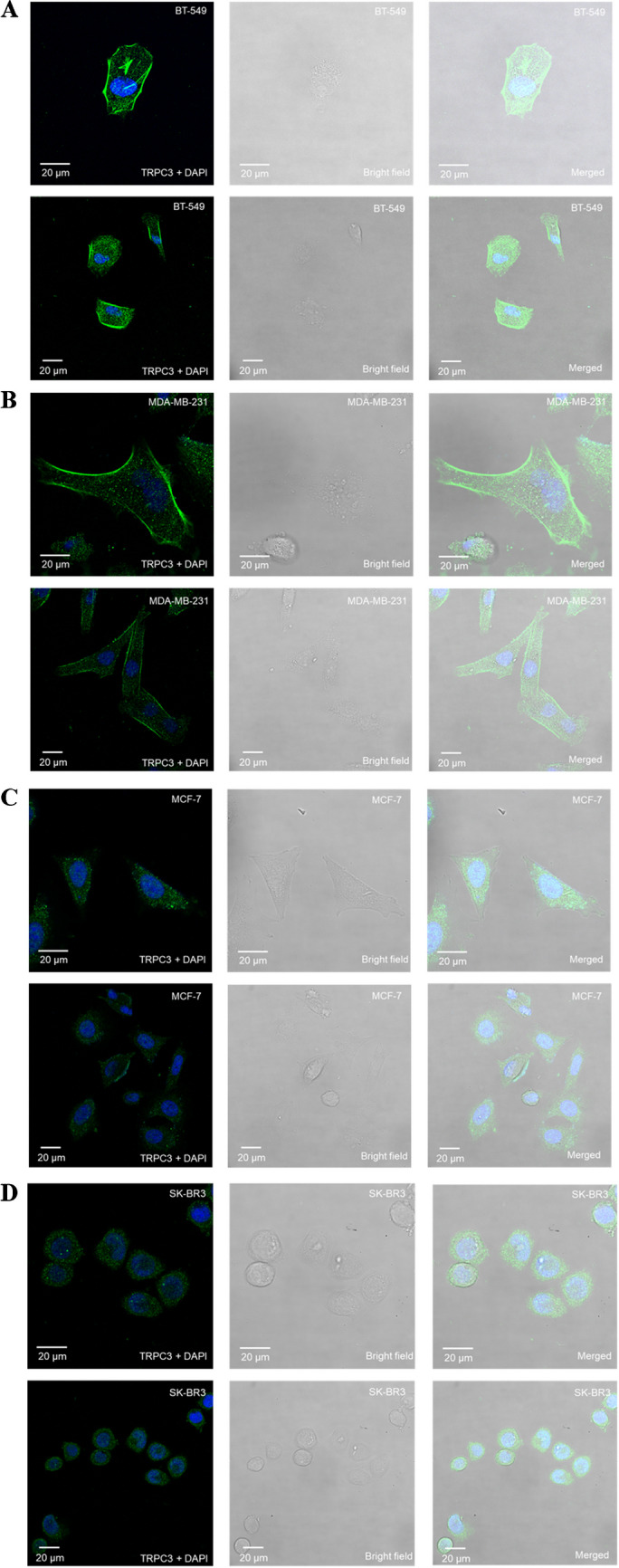
TNBC expresses TRPC3 on plasma membrane. Representative confocal images showing the subcellular localization of TRPC3 (green) in four different breast cancer cell lines. Cells were stained with a TRPC3 antibody and DAPI (blue). Merging fluorescence images with bright field images showed that TRPC3 was highly expressed in the plasma membrane of the two TNBC lines, **A** BT-549 and **B** MDA-MB-231, when compared to **C** ER + cell line MCF-7 and **D** HER + cell line SK-BR3. Results were representative of three independent experiments. Scale bar: 20 μm

**Fig. 2:**
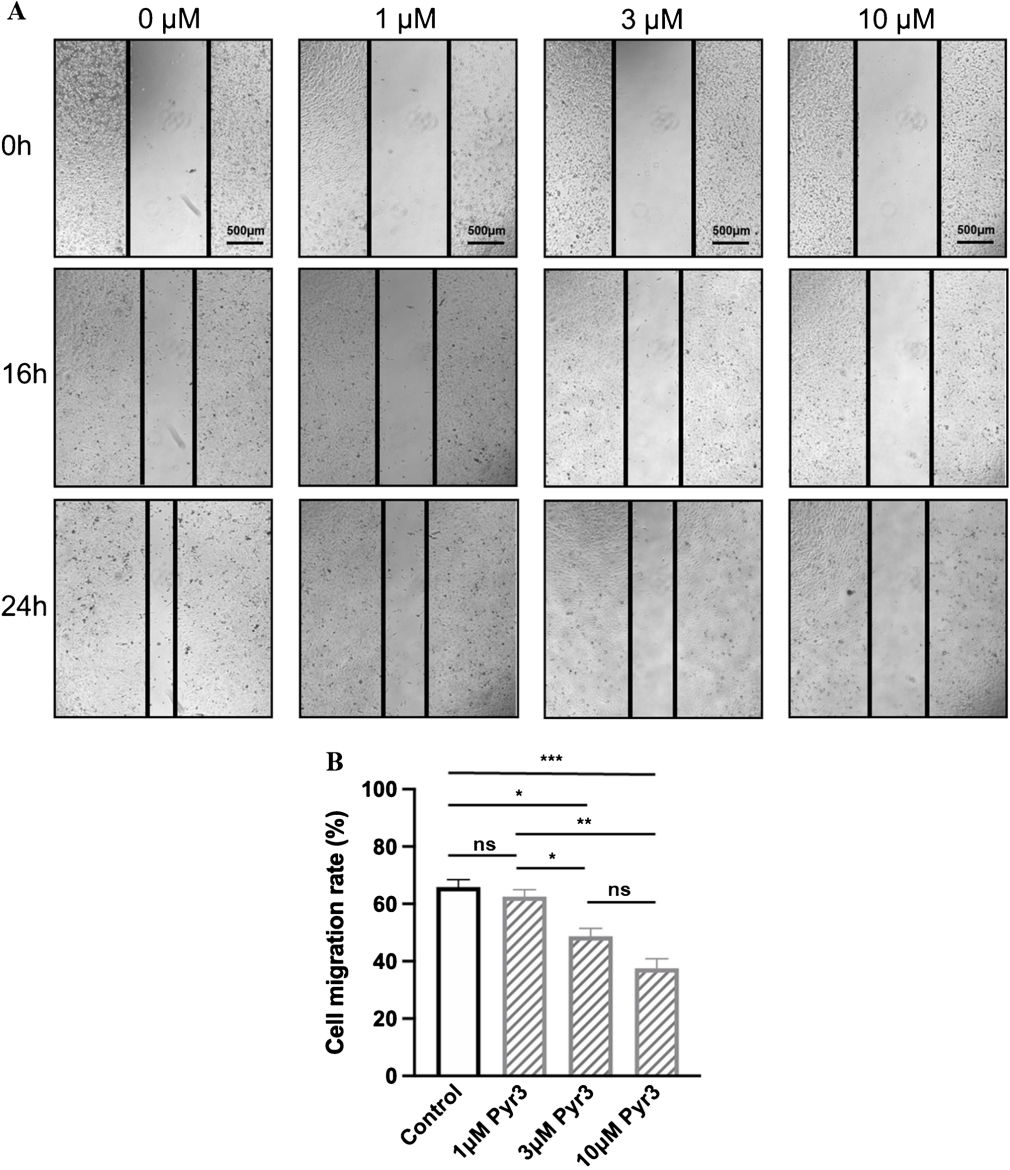
Blockade of TRPC3 decreased migration of TNBC cells BT-549.** A** Representative images showing the wound gap at 0 h, 16 h and 24 h after wound scratch in the wound healing assay under treatments of different concentrations of Pyr3, the pharmacological blocker of TRPC3. Scale bar: 500 μm. **B** Summarized data on the cell migration rates 24 h after wound scratch. Cell migration rates in the 3 μM and 10 μM Pyr3 treated groups were significantly decreased at 24 h when compared to DMSO control. Values are mean ± SEM (*n* = 3). * *P* < 0.05, ** *P* < 0.01, *** *P* < 0.001

**Fig. 3:**
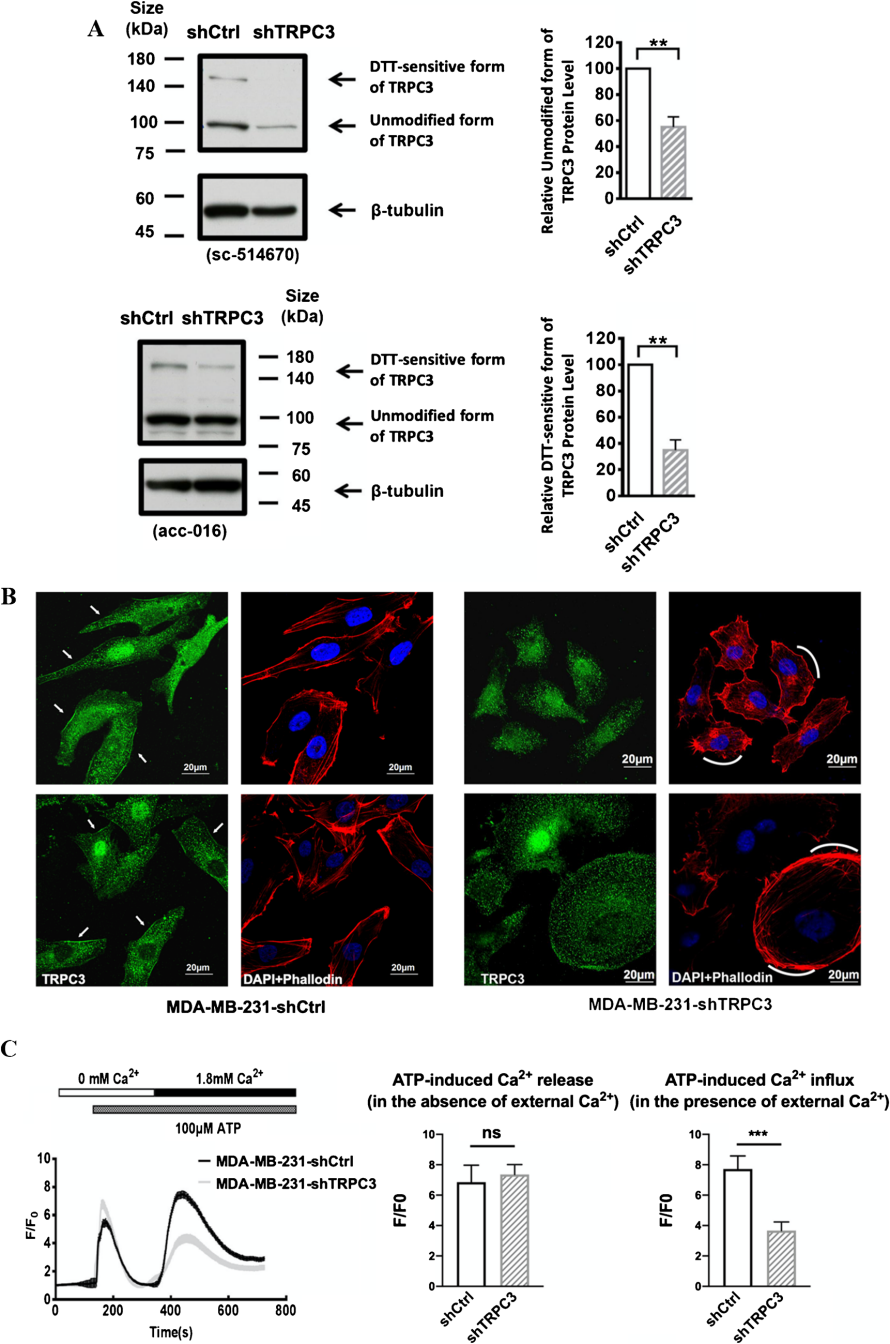

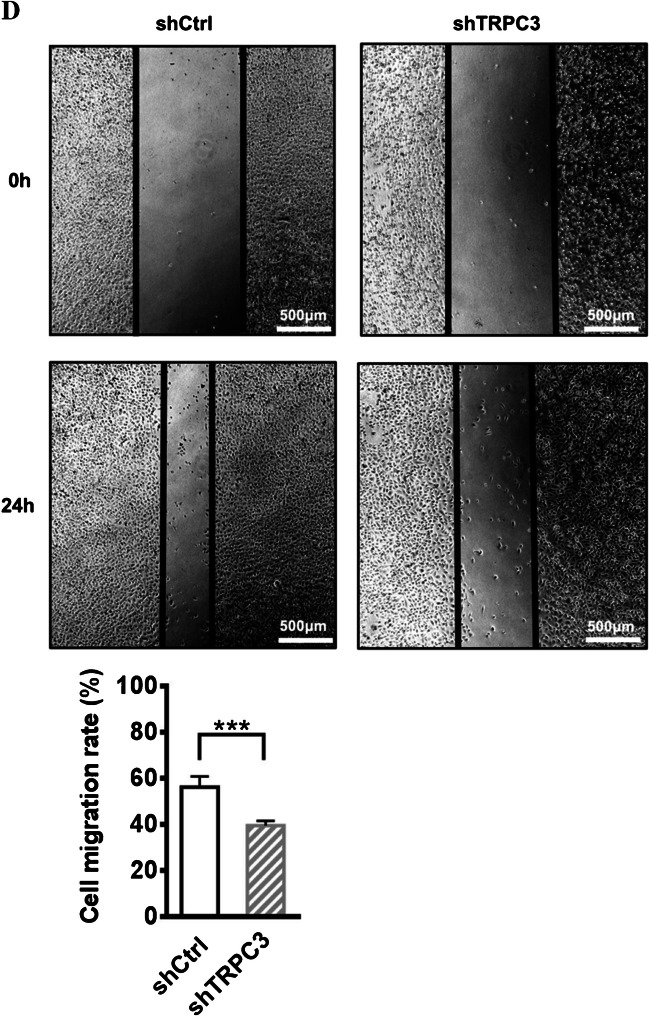
Long-term TRPC3 inhibition decreased the migration of TNBC cells MDA-MB-231.** A**-**C** MDA-MB-231 cell line with long-term knockdown of TRPC3 expression and function was successfully made. **A** (Left) Representative Western blots showing that the expression of both the unmodified form of TRPC3 protein (the band between 75 and 100 kDa) and the dithiothreitol (DTT)-sensitive form of TRPC3 protein (the band between 140 and 180 kDa) in MDA-MB-231 cells transduced with shTRPC3 (MDA-MB-231-shTRPC3) and in MDA-MB-231 cells transduced with shCtrl (MDA-MB-231-shCtrl; as control). Two different anti-TRPC3 antibodies (sc-514670, acc-016) were used and consistent results were obtained. The heavier band was also found to be DTT-sensitive in our previous study [44]. β-tubulin was used as a loading control. (Right) Summarized data on TRPC3 expression using data from santa cruz sc-514670 antibodies. TRPC3 expression level was firstly normalized to β-tubulin expression level and the values in each group were then normalized to that of the shCtrl group. The expression of both the unmodified form and the DTT-sensitive form of TRPC3 proteins were reduced in MDA-MB-231-shTRPC3 when compared to the control MDA-MB-231-shCtrl. Values are mean ± SEM (*n* = 3). ** *P* < 0.01. **B** Representative confocal images showing the expression of TRPC3 (green) by anti-TRPC3 and cytoskeletal protein actin (red) by phalloidin in MDA-MB-231-shCtrl and MDA-MB-231-shTRPC3 cells. TRPC3 expression in the plasma membrane of (right) MDA-MB-231-shTRPC3 cells was down-regulated when compared to that of (left) control MDA-MB-231-shCtrl cells. Plasma membrane positions were indicated by white arrows. MDA-MB-231-shTRPC3 cells were also different from control cells as revealed by cell morphology and actin staining. (Left) Most of MDA-MB-231-shCtrl cells exhibited quite elongated cortical F-actin (red) patterns with bifurcated extensions (as indicated by double-sided white arrows). (Right) In contrast, contractile cell morphology was observed in those MDA-MB-231-shTRPC3 cells with smaller cell size; MDA-MB-231-shTRPC3 with larger cell size displayed an increased dense meshwork of unpolarized actin filaments around the cell periphery (as indicated by white curves). Nuclei were stained by DAPI. Results were representative of three independent experiments. Scale bar: 20 μm. **C** (Left) Average Ca^2+^ imaging trace showing changes in the level of cytosolic free Ca^2+^ over time in MDA-MB-231-shTRPC3 cells and control MDA-MB-231-shCtrl cells. Average fluo-4 fluorescence intensity was transiently increased in response to 100 μM ATP when external Ca^2+^ was absent. Addition of external calcium (1.8 mM) led to a differential increase in fluorescence intensity in MDA-MB-231-shTRPC3 and control MDA-MB-231-shCtrl cells. (Middle—Right) Summarized data of fluorescence (F) normalized to baseline fluorescence (F0) (F/F0) for (middle) ATP-induced Ca^2+^ release in the absence of external Ca^2+^ and for (right) ATP-induced Ca^2+^ influx in the presence of external Ca^2+^. ATP-induced Ca^2+^ influx amplitude in MDA-MB-231-shTRPC3 cells was significantly reduced when compared to that of MDA-MB-231-shCtrl cells. Traces of fluorescence intensity are average of 50–80 cells measured in total from three independent experiments. Values are mean ± SEM (*n* = 3). *** *P* < 0.001. n.s.: not significant. **D** (Upper) Representative images captured by phase-contrast microscope showing the wound gap at 0 and 24 h after wound scratch in the wound healing assay. Scale bar: 500 μm. (Lower) Summarized data on the cell migration rate. Cell migration rate of MDA-MB-231-shTRPC3 was significantly decreased at 24 h when compared to that of MDA-MB-231-shCtrl. Values are mean ± SEM (*n* = 3). *** *P* < 0.001. n.s.: not significant

Immunostaining followed by confocal microscopy revealed that TNBC cell lines MDA-MB-231 and BT-549 expressed TRPC3 in their plasma membrane, unlike ER^+^ line MCF-7 and HER^2+^ line SK-BR3 (Fig. 1), consistent with our previous report [44]. To investigate the role of TRPC3 in the migration of TNBC cells, Pyr3, the pharmacological blocker of TRPC3, was firstly used to decrease the activity of TRPC3. Pyr3 was found to decrease the migration of BT-549 in a concentration-dependent manner (Fig. 2). On the other hand, MCF-7 and SK-BR3 cells, which express only a low level of TRPC3, were insensitive to Pyr3 (Supplementary Fig. 1). In addition, lentiviral vector harboring shRNA-TRPC3 (lenti-shTRPC3) was used to knockdown TRPC3 in long run in MDA-MB-231. Lentiviral vector harboring non-targeting control shRNA (shCtrl) was used as control. After transduction with lentiviruses and selection by antibiotics, stable cell lines MDA-MB-231-shCtrl and MDA-MB-231-shTRPC3 were established (Figs. 3A – B). Expectedly, migration of untransfected MDA-MB-231 cells did not differ from that of MDA-MB-231 cells transfected with lenti-shCtrl (Supplementary Fig. 2). Worth to note, heterogeneous changes of cell morphology were observed in MDA-MB-231-shTRPC3 group. Redistribution of F-actin was detected by immunofluorescent staining with Alexa-568-conjugated phalloidin followed by confocal imaging. Most of MDA-MB-231-shCtrl cells exhibited quite elongated cortical F-actin patterns with bifurcated extensions (Fig. 3B, left panel). In contrast, contractile cell morphology was observed in those MDA-MB-231-shTRPC3 cells with smaller cell size (Fig. 3B, upper right panel); at the same time, MDA-MB-231-shTRPC3 with larger cell size displayed an increased dense meshwork of unpolarized actin filaments around the cell periphery (Fig. 3B, lower right panel). At functional level, knockdown of TRPC3 decreased the basal level of Ca^2+^ (Supplementary Fig. 3). In addition, TRPC3 knockdown was found to inhibit Ca^2+^ influx in MDA-MB-231 (Fig. 3C). In the presence of Ca^2+^-free external solution, no statistical difference was found in the ATP-induced calcium release (F/F0) between the MDA-MB-231-shCtrl and MDA-MB-231-shTRPC3 cells. After re-supplying cells with 1.8 mM Ca^2+^-containing external solution, calcium influx was significantly reduced in MDA-MB-231-shTRPC3 when compared to MDA-MB-231-shCtrl (Fig. 3C). All of these data suggested that stable cell lines MDA-MB-231-shCtrl and MDA-MB-231-shTRPC3 were successfully established and that we had novel tools for investigating the function of long-term knockdown of TRPC3 in TNBC.

To investigate the effect of TRPC3 knockdown on cell migration, wound healing assay was performed. 24 h after wound scratching, gap closing rate of scratch wounds was significantly decreased in MDA-MB-231-shTRPC3 when compared to MDA-MB-231-shCtrl (Fig. 3D). Altogether, decrease in TRPC3 function was found to inhibit the migration of TNBC.

### TRPC3 regulated the nuclear translocation of calcium-sensitive transcription factor NFATc1 in MDA-MB-231

**Fig. 4:**
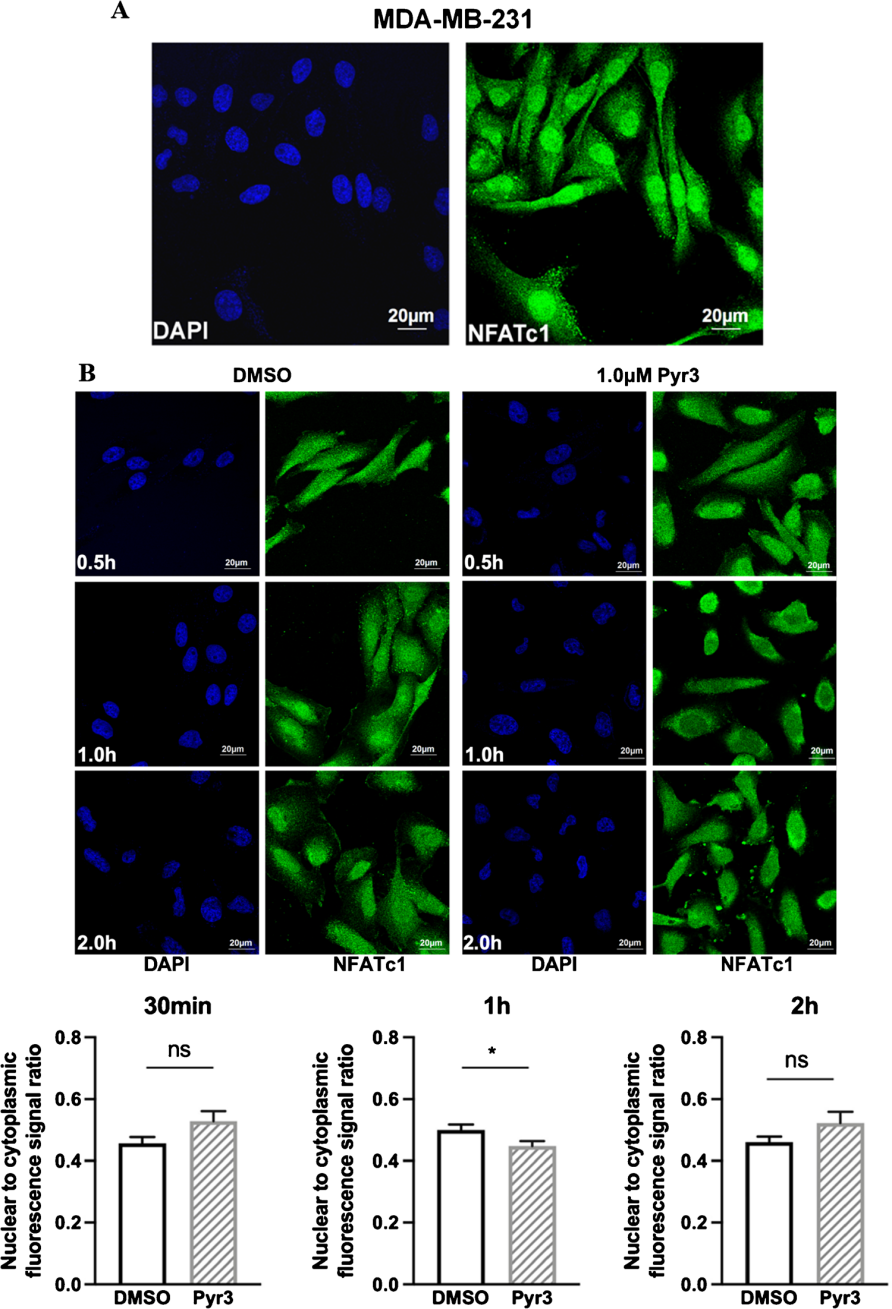

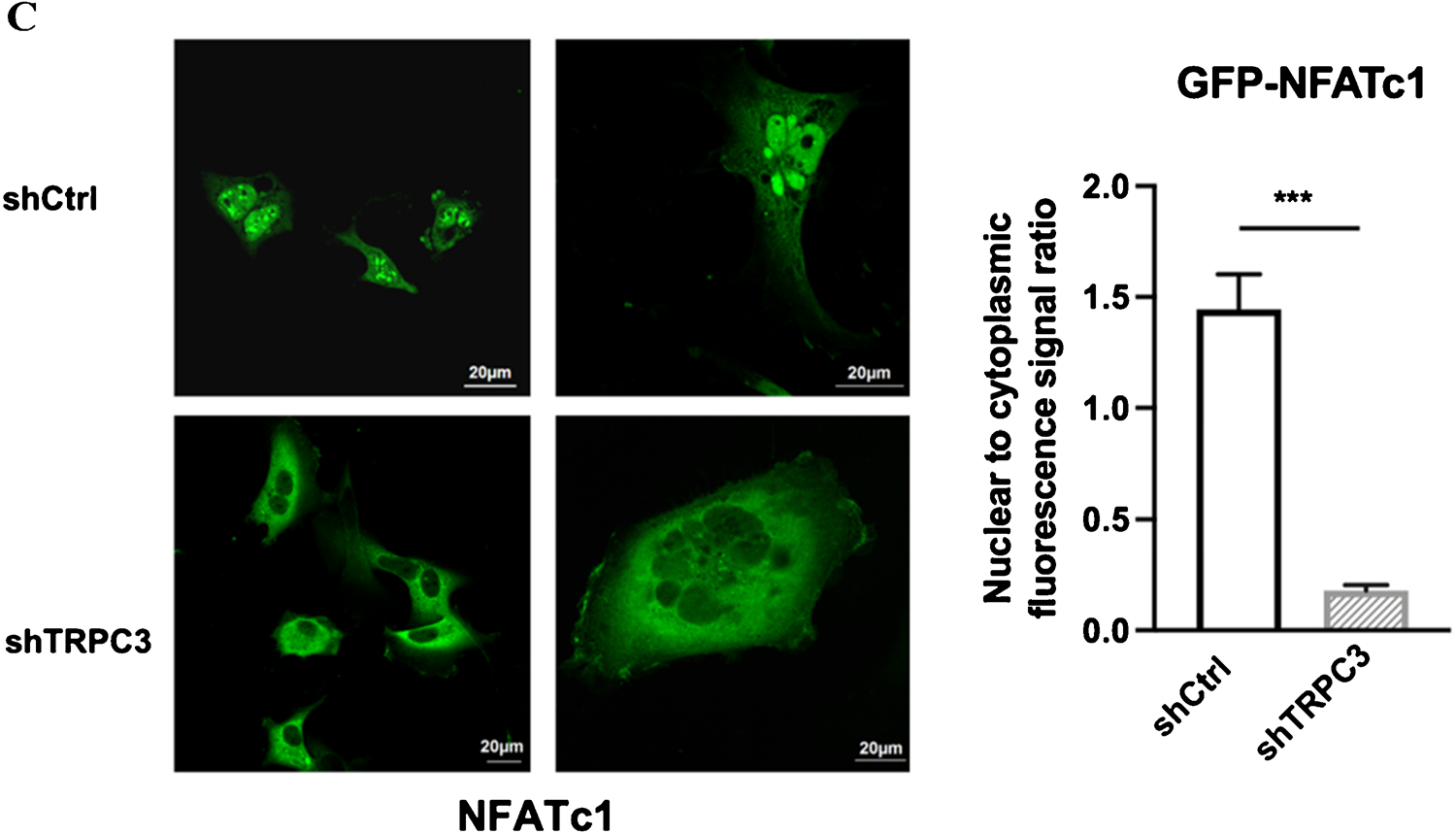
TRPC3 positively regulated the calcium-sensitive transcription factor NFATc1 in MDA-MB-231 cells.** A**-**B** Representative confocal images showing the subcellular localization of NFATc1 in different groups. NFATc1 (green) was stained with NFATc1 antibody, and nuclei were stained with DAPI (blue). **A** In MDA-MB-231, nuclear NFATc1 staining was strong, suggesting that NFATc1 is constitutively active in MDA-MB-231. **B** (Upper) Representative confocal images showing the subcellular localization of NFATc1 in MDA-MB-231 at specific time points upon TRPC3 blockage by Pyr3. Blocking TRPC3 induced NFATc1 translocation from nucleus to cytoplasm in MDA-MB-231 cells. Scale bar: 20 μm. (Lower) Summarized data on the nuclear-to-cytoplasmic fluorescence signal ratio. TRPC3 blockage for 1.0 h significantly decreased the nuclear/cytoplasmic ratio of NFATc1. Values are mean ± SEM (*n* = 3), with 40–60 cells measured in total. * *P* < 0.05. n.s.: not significant. **C** (Left) Representative confocal images showing MDA-MB-231-shCtrl and MDA-MB-231-shTRPC3 cells transfected with GFP-NFATc1. Less signal from GFP-NFATc1 was observed within the nucleus in MDA-MB-231-shTRPC3 cells when compared to that in MDA-MB-231-shCtrl cells. Scale bar: 20 μm. (Right) Summarized data on the nuclear-to-cytoplasmic fluorescence signal ratio. Knockdown of TRPC3 in MDA-MB-231-shTRPC3 caused a lower nuclear/cytoplasmic ratio of NFATc1 when compared to that in control MDA-MB-231-shCtrl cells. Values are mean ± SEM (*n* = 3), with 50–80 cells measured in total. *** *P* < 0.001

Endogenous NFATc1 expression in MDA-MB-231 was detected by immunocytochemistry followed by confocal imaging. MDA-MB-231 have constitutively activated nuclear NFATc1 (Fig. 4A). Blocking TRPC3 by Pyr3 transiently decreased the nuclear-to-cytoplasmic fluorescence signal ratio of NFATc1 at the time point of 1.0 h when compared to the DMSO control group (Fig. 4B). To further verify the effect of TRPC3 blockade on the translocation of NFATc1, GFP-NFATc1 plasmid was transfected into MDA-MB-231-shCtrl and MDA-MB-231-shTRPC3 and the green fluorescence signal was observed. In MDA-MB-231-shCtrl, the green fluorescence signal in the nucleus was significantly higher than that in the cytoplasm (Fig. 4C). In contrast, in MDA-MB-231-shTRPC3, the green fluorescence signal mainly localized to the cytoplasm. These results suggested that blocking TRPC3 and knockdown of TRPC3 increased the translocation of NFATc1 from nucleus to cytoplasm. We concluded that TRPC3 was upstream of NFATc1 pathway in MDA-MB-231.

### Knockdown of TRPC3 down-regulated glypican-6; NFATc1, of which nuclear translocation was regulated by TRPC3, regulated glypican-6 expression

**Fig. 5:**
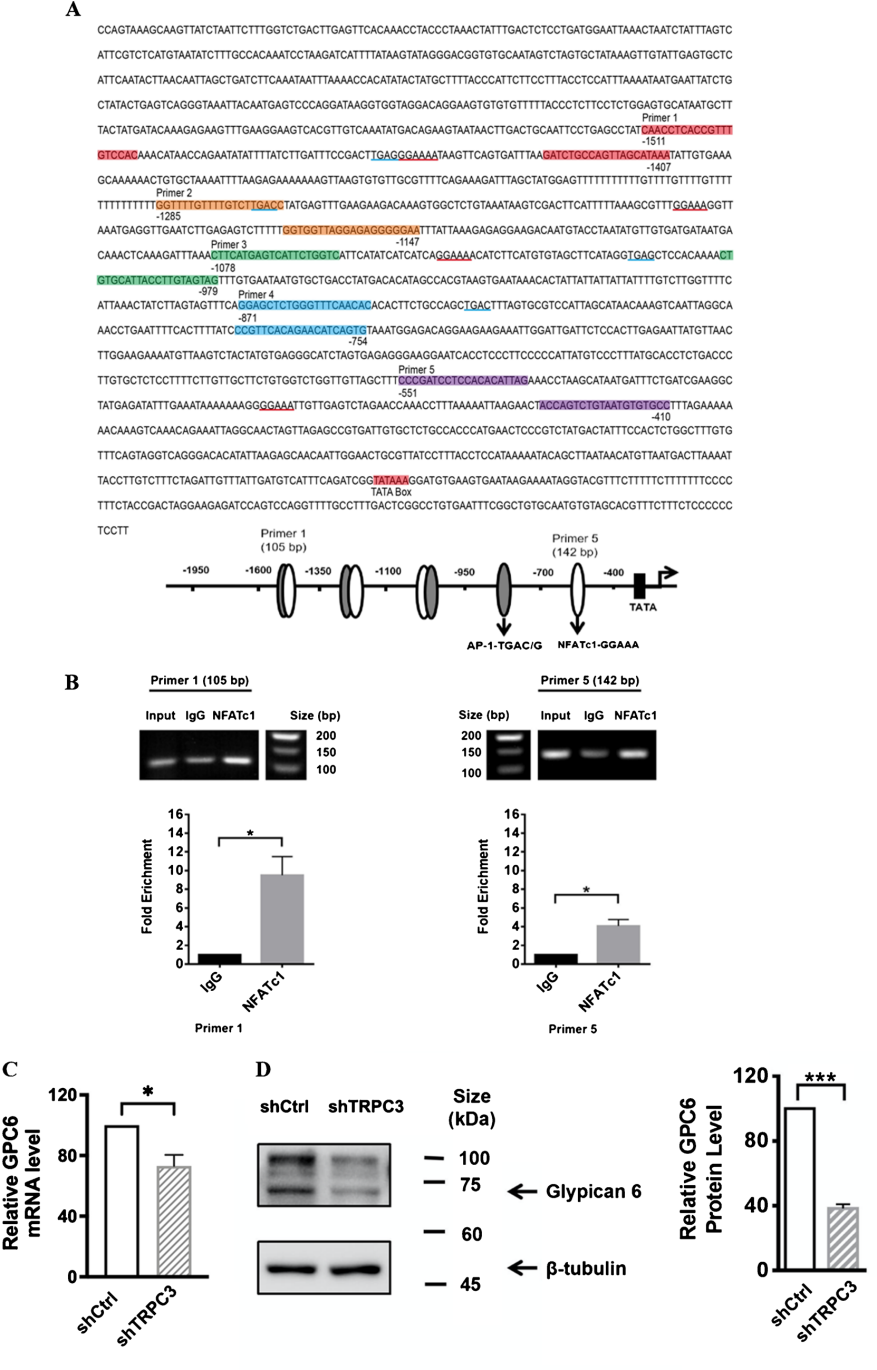
Glypican-6 (GPC6) is a transcription target of NFATc1 expression in MDA-MB-231 and knockdown of TRPC3 down-regulated GPC6.** A** (Upper) Nucleotide sequence of the human GPC6 putative promoter region from − 2000 to + 2. The putative NFATc1/AP-1 binding sites were underlined. The numbers indicate the targeting positions relative to the transcriptional start site. (Lower) Schematic analysis of NFATc1 and AP-1 protein binding sites at the GPC6 promoter. **B** (Upper) Stronger intensity of bands representing GPC6 promoter regions (-1511 to -1407) and (-551 to -410) in NFATc1-precipitated DNA; qPCR products were loaded in the same amount and visualized through agarose gel electrophoresis. (Lower) Summarized data showing increased enrichment of GPC6 promoter regions (-1511 to -1407) and (-551 to -410) in NFATc1-precipitated DNA when compared with IgG-precipitated DNA (control group). Values are mean ± SEM (*n* = 3). * *P* < 0.05. **C—D** Long-term knockdown of TRPC3 down-regulated GPC6 in MDA-MB-231. **C** GPC6 expression at mRNA level in MDA-MB-231-shTRPC3 cells and MDA-MB-231-shCtrl cells. Values are mean ± SEM (*n* = 3). * *P* < 0.05. **D** (Left) Representative Western blots showing the expression of GPC6 in MDA-MB-231-shTRPC3 cells and MDA-MB-231-shCtrl cells. β-tubulin was used as a loading control. (Right) Summarized data on GPC6 protein expressions. GPC6 expression level was firstly normalized to β-tubulin expression level and the values in each group were then normalized to that of the shCtrl group. Knockdown of TRPC3 decreased the expression of GPC6. Values are mean ± SEM (**n** = 3). * *P* < 0.01, *** *P* < 0.001

Glypican (GPC)-6 (GPC6) was reported to modulate invasive migration in breast cancer cells and was transcriptionally regulated by NFATc2 [48]. Based on these previous findings, we speculated that GPC6 may be a potential intermediate player downstream of NFATc1 signaling in modulating MDA-MB-231 cell migration. To determine whether GPC6 is a direct NFATc1 target gene in MDA-MB-231, ChIP assay was performed. Previous studies reported that NFAT minimal consensus sequence is GGAAA(A), while AP-1 minimal consensus sequence is TGAC/G [18, 35]. Sequence scanning of the GPC6 promoter revealed four NFATc1 and four AP-1 protein-binding sites upstream of the transcriptional start site (Fig. 5A). The protein-DNA complexes were immunoprecipitated with either anti-NFATc1 or anti-IgG antibody. We identified two regions in the GPC6 promoter as potential binding sites of NFATc1 in MDA-MB-231. Enrichment of NFATc1 binding to the GPC6 promoter region (-1511 to -1407) and (-551 to -410) was observed by qPCR analysis of the precipitated DNA, followed by visualization of the qPCR products through agarose gel electrophoresis (Fig. 5B). As TRPC3 is upstream of NFATc1 in MDA-MB-231 and NFATc1 binds to the promoter of GPC6, we inferred that long-term knockdown of TRPC3 would affect GPC6 expression. Consistently, GPC6 was found to be down-regulated in MDA-MB-231-shTRPC3 when compared to the MDA-MB-231-shCtrl at both mRNA (Fig. 5C) and protein levels (Fig. 5D).

### Glypican-6 regulated MDA-MB-231 cell migration through the interaction with vinculin

**Fig. 6:**
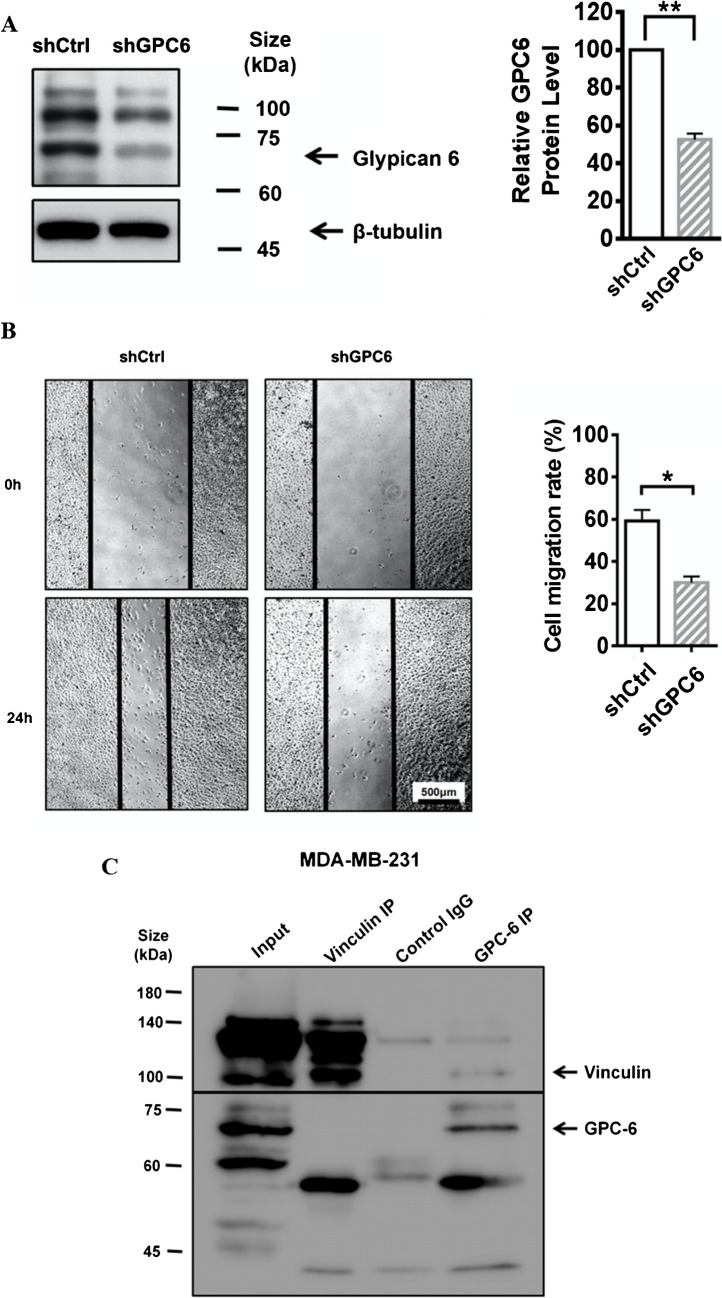

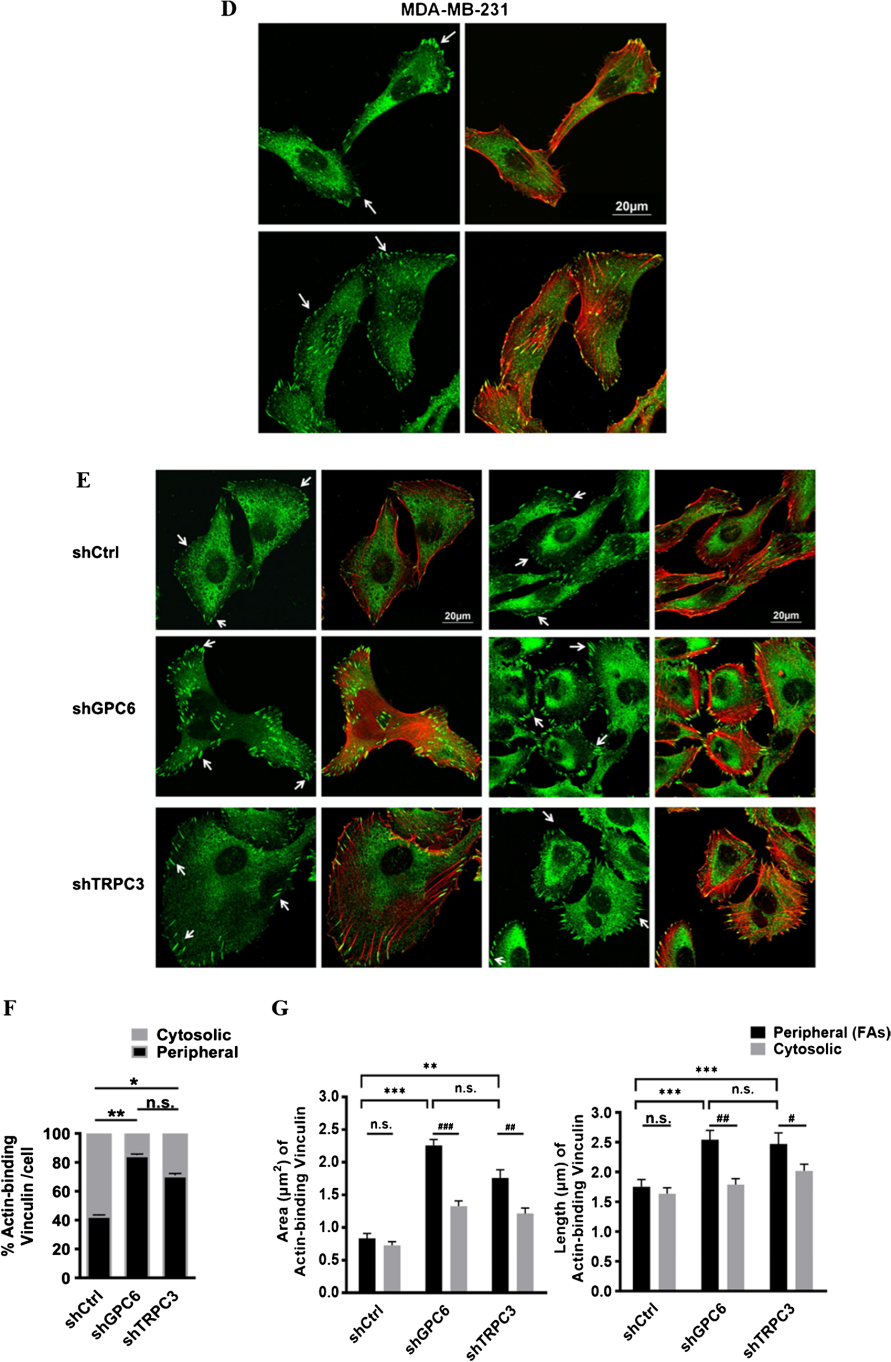
GPC6 positively regulated the migration of MDA-MB-231.** A** (Left) Representative Western blots showing that lentiviral vector harboring shRNA-GPC6 down-regulated the protein expression level of GPC6 in MDA-MB-231 cells. β-tubulin was used as a loading control. (Right) Summarized data on GPC6 expression. GPC6 expression level was firstly normalized to β-tubulin expression level and the values in each group were then normalized to that of the shCtrl group. The data indicated that MDA-MB-231 cells with long-term knockdown of GPC6 (MDA-MB-231-shGPC6) were successfully made. Values are mean ± SEM (*n* = 3). ** *P* < 0.01. **B** (Left) Representative images captured by phase-contrast microscope showing the wound gap at 0 and 24 h after wound scratch in the wound healing assay. Scale bar: 500 μm. (Right) Summarized data on the cell migration rate. Cell migration rate of MDA-MB-231-shGPC6 cells was significantly decreased at 24 h when compared to that of MDA-MB-231-shCtrl cells. Values are mean ± SEM (*n* = 3). * *P* < 0.05. **C** Representative Western blots showing the interaction between GPC6 and vinculin as revealed by Co-IP assay. MDA-MB-231 cell lysate was subjected to immunoprecipitation (IP) with control IgG (mouse), anti-vinculin or anti-GPC6 antibodies. Co-immuoprecipitated proteins were visualized in Western blots. Membranes were incubated with anti-vinculin and anti-GPC6 antibodies. Vinculin (~ 116 kDa) was detected in GPC6 (~ 62 kDa) IP samples. Results were representative of three independent experiments. **D**-**E** Representative confocal images showing the cell morphology, actin cytoskeleton organization and assembly of focal adhesions (FAs) in different groups. Fixed cells were stained with anti-vinculin (green) and phalloidin (to indicate actin; red). FAs were indicated by white arrows. MDA-MB-231-shTRPC3 and MDA-MB-231-shGPC6 both displayed an increased dense meshwork of cortical actin bundles around the cell periphery when compared to MDA-MB-231-shCtrl. Scale bar: 20 μm. **F** Summarized data on the percentage of counts with actin-binding vinculin for those in the cytosol (grey bar) and in the cell periphery (black bar) to the total counts per cell. Knockdown of GPC6 or TRPC3 both increased the percentage of peripheral FAs in MDA-MB-231 cells. Values are mean ± SEM (*n* = 3). * *P* < 0.05. ** *P* < 0.01. n.s.: not significant. **G** Summarized data on the (left) area and (right) length of co-localized vinculin and actin. Both the area and the length of peripheral actin-binding vinculin (FAs) of MDA-MB-231-shGPC6 and MDA-MB-231-shTRPC3 were larger than those of MDA-MB-231-shCtrl cells. In addition, the area and the length of peripheral FAs were larger than those of cytosolic actin-binding vinculin in MDA-MB-231-shGPC6 and MDA-MB-231-shTRPC3 cells while such was not observed in MDA-MB-231-shCtrl cells. The area and length of FAs were measured using the NIH Image J software. Values are mean ± SEM (*n* = 3, from a total of > 400 FAs). * *P* < 0.05; ** *P* < 0.01; *** *P* < 0.001 (among different experimental groups); # *P* < 0.05; ## *P* < 0.01; ### *P* < 0.001 (among peripheral and cytosolic data)

To investigate the role of GPC6 in MDA-MB-231, we established the stable cell line MDA-MB-231-shGPC6 using lentivirus harboring shRNA-GPC6. Decreased GPC6 protein expression level examined by Western blots validated the specificity of shGPC6 in MDA-MB-231 (Fig. 6A). MDA-MB-231-shGPC6 cell migration rate was determined by wound healing assay. 24 h after wound scratching, similar to MDA-MB-231-shTRPC3, MDA-MB-231-shGPC6 showed a significant decrease in cell migration when compared to MDA-MB-231-shCtrl (Fig. 6B). Our results suggested that GPC6 is positively correlated with the migration of MDA-MB-231.

The underlying mechanism of how GPC6 regulates cell migration in MDA-MB-231 was further examined. Peripheral FAs with higher activities are known to be required for cell migration [9]. However, FA-stabilizing vinculin with increased size were reported to impair directed cell migration [47]. Based on the phenotype of the actin cytoskeleton reorganization in MDA-MB-231-shTRPC3 cells, we moved to test the hypothesis that formation of peripheral FAs with impaired migration (as indicated by peripheral actin-binding vinculin [9]) would be increased due to TRPC3 suppression.

Co-immunoprecipitation (Co-IP) followed by western blotting was performed to test if there is physical interaction between GPC6 and vinculin. Interestingly, vinculin (~ 116 kDa) was detected in GPC6 (~ 62 kDa) IP samples (Fig. 6C). Reversely, GPC6 detected in vinculin IP samples was extremely weak. Previous studies showed that there are different cytosolic and plasma membrane vinculin and meta-vinculin isoforms expressed in MDA-MB-231 cells [5]. Therefore, it is possible that the specific form of vinculin that interacts with GPC6 was not pulled down in a significant amount. Since vinculin is a major member of FA proteins, the interaction between GPC6 and vinculin guided us to further look at the change of peripheral FAs due to GPC6 suppression and TRPC3 suppression. Endogenous vinculin expression in MDA-MB-231 was detected by immunocytochemistry followed by confocal imaging. In non-stimulated MDA-MB-231 cells, vinculin was organized on the leading edge of F-actin and presented in small dot-like forms (Fig. 6D). These small dot-like vinculin proteins expressed at the cell periphery indicate the early stage of FA formation [9]. On the other hand, MDA-MB-231-shTRPC3 and MDA-MB-231-shGPC6 both displayed an increased dense meshwork of unpolarized actin filaments around the cell periphery (Fig. 6E). Percentage of peripheral actin-binding vinculin to the total vinculin counts/cell was remarkably increased in MDA-MB-231-shTRPC3 and MDA-MB-231-shGPC6 when compared to MDA-MB-231-shCtrl (Fig. 6F). Indeed, similarly, Pyr3, the pharmacological blocker of TRPC3, increased the percentage of peripheral actin-binding vinculin to the total vinculin counts/cell in another TNBC line BT549 (Supplementary Fig. 4). The results suggested that GPC6 suppression or TRPC3 suppression increased the peripheral actin-binding vinculin formation in TNBC cells. Streak-like vinculin located at the cell periphery of MDA-MB-231-shTRPC3 and MDA-MB-231-shGPC6 indicated the assembly of FAs [9]. Interestingly, a significant increase in both the area and length of peripheral actin-binding FAs were shown in MDA-MB-231-shTRPC3 and MDA-MB-231-shGPC6 when compared to MDA-MB-231-shCtrl (Fig. 6G). Moreover, both the area and length of peripheral actin-binding vinculin were increased when compared with those of cytosolic actin-binding vinculin in MDA-MB-231-shTRPC3 and MDA-MB-231-shGPC6, while there was no difference between the two in MDA-MB-231-shCtrl (Fig. 6G). Similar results were obtained when Pyr3 was applied to BT549 (Supplementary Fig. 4). Altogether, these results suggested that decreasing the function/ expression of GPC6 and TRPC3 consistently induced a larger, elongated actin-bound peripheral FAs in TNBC cells.

## Discussion

**Fig. 7:**
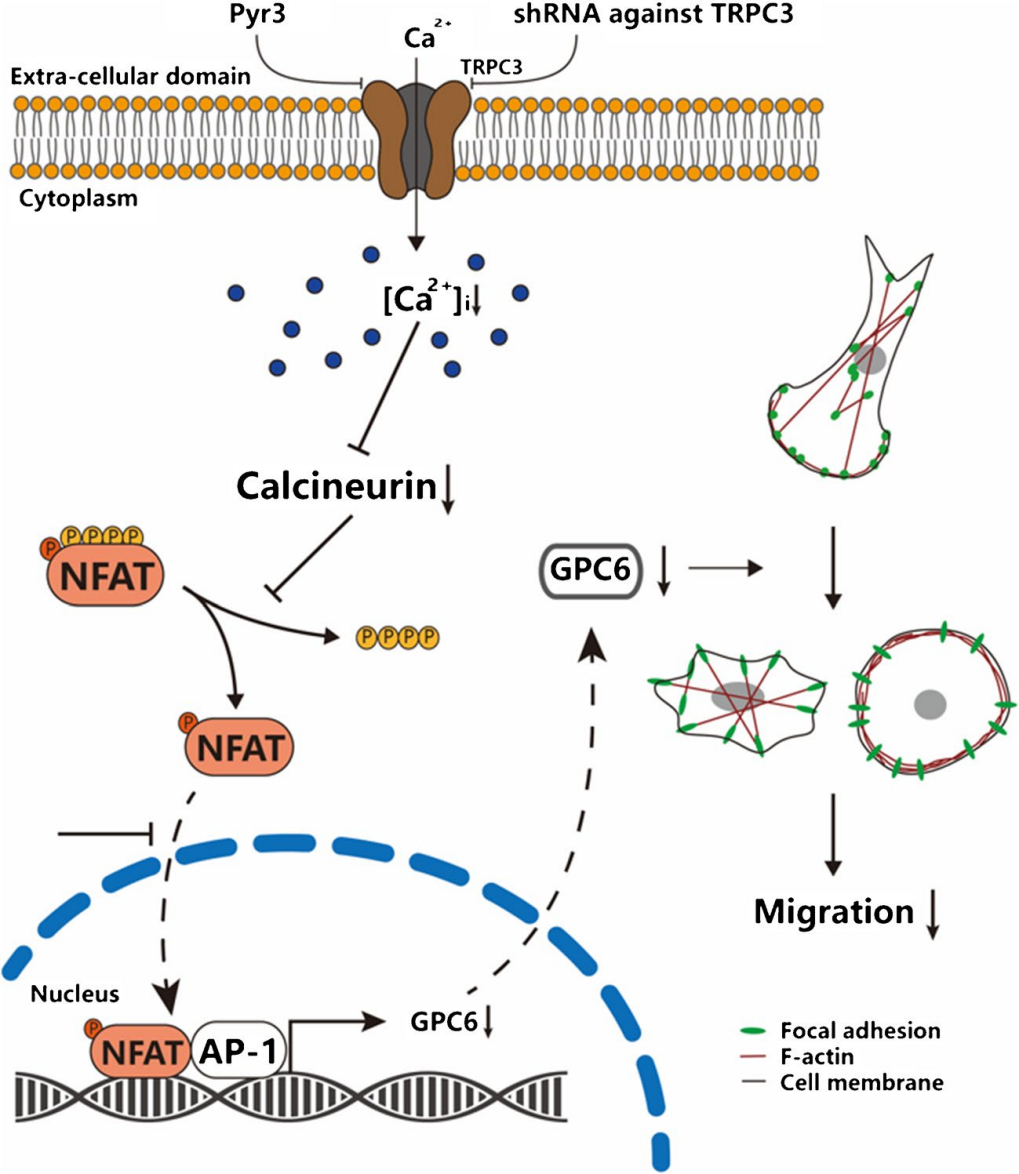
A schematic diagram explaining how TRPC3 and NFATc1 signaling would regulate cell migration in MDA-MB-231. Long-term knockdown of TRPC3 (e.g. by lenti-shTRPC3) inhibits the Ca2 + entry across plasma membrane. This decrease in Ca2 + influx would in turn cause the translocation of NFATc1 from the nucleus to the cytosol and the NFATc1 signaling pathway would be inactivated concomitantly. When NFATc1 is activated, it would bind to the promoter of GPC6 and positively affect its expression. Due to the translocation of NFATc1 to the cytosol, nuclear NFATc1 would decrease, leading to less GPC6 being transcribed and expressed. In MDA-MB-231, GPC6 proteins directly interact with vinculin (a component of FAs) and regulate the actin cytoskeleton organization and the dynamics of FAs turnover. These would in turn determine cell migration. Long-term knockdown of GPC6 decreases migration of MDA-MB-231. Our study highlights a key functional role of the TRPC3-NFATc1-GPC6-vinculin signaling cascade in maintaining the migration of TNBC cells

The major novel findings of this study are as follows: (1) decreasing the activity and/or the expression of TRPC3 decreases the migration of TNBC cells; (2) the activity of TRPC3 determines the subcellular translocation of NFATc1; (3) GPC6 is a transcriptional target of NFATc1 in TNBC cells; (4) GPC6 is down-regulated in TNBC cells in response to TRPC3 suppression; (5) GPC6 directly interacts with vinculin in TNBC cells; (6) decreasing the activity and/or knockdown of TRPC3 and GPC6 decrease the migration of TNBC cells probably through changing actin cytoskeleton organization and decreasing the dynamics of FA turnover. Taken all these findings together, we highlight a key functional role of the TRPC3-NFATc1-GPC6-vinculin signaling cascade in maintaining the migration of TNBC cells. A schematic illustration is shown in Fig. 7.

TRPC3 has previously been reported to be upregulated in breast cancer biopsy [2]. Using pharmacological blocker and transient expression of the dominant negative construct of TRPC3, we have previously reported that TRPC3 channels positively modulate cell proliferation and apoptosis resistance of TNBC MDA-MB-231 cells. In the present study, long term knockdown of TRPC3 was found to decrease the migration ability of TNBC cells. In fact, other TRP proteins were also found to be involved in multiple cellular processes. For instance, a decreased level of TRPC6 in MDA-MB-231 cells impaired the proliferation and migration of the cells by regulating calcium entry [14]. To add some complexities to the problem, TRPC3 and TRPC6 have been reported to form heterodimers in some cells such as human embryonic kidney 293 (HEK293) cells [41]. There is evidence suggesting that downregulation of TRPC3 could lead to a compensatory upregulation of TRPC6 [13]. Some studies suggested that TRPC6 is highly homologous to TRPC3 [38], with potentially redundant functions [1]. On the other hand, some previous studies have also demonstrated that for TRPC channel proteins forming heterodimers, the downregulation of one protein does not necessarily result in a compensatory increase in the other [6]. It is also possible that these channels are co-regulated, and TRPC3 knockdown may lead to a concurrent decrease in TRPC6 levels. While the downstream consequences of TRPC3 knockdown can be determined by other experiments such as whole transcriptome sequencing, the observed differences between the TRPC3 knockdown cell line and the control cell line in our current study are primarily attributed to the downregulation of TRPC3. To the best of our knowledge, our study is the first to knockdown TRPC3 using shRNAs in breast cancer to explore its long-term role in TNBC migration.

According to the previous study, strong nuclear NFATc1 staining was detected in TNBC tissues [34]. Consistently, we found that NFATc1 was mainly localized in the nucleus of MDA-MB-231. Thus, we focused on the NFATc1 signaling pathway activated in MDA-MB-231. Interestingly, we found that blocking TRPC3 induced NFATc1 translocation from nucleus to cytoplasm in MDA-MB-231, hinting that TRPC3 suppression may inhibit the activation of NFATc1 through blocking calcium entry. Previous studies showed that TRP channels-mediated calcium signaling plays a critical functional role in cancer cell progression [31, 37]. For instance, TRPC5-mediated Ca^2+^ entry was reported to stimulate P-glycoprotein production in adriamycin-resistant MCF-7/ADM breast cancer cells via NFATc3 [23]. To comprehensively understand the signaling cascade regulated by TRPC3, finding out the targeted gene transcribed by NFATc1 is crucial. GPC6 is a member of GPC family of thiohepatic glycoproteins [30]. GPC6, which was down-regulated in MDA-MB-231-shTRPC3, was previously reported to be transcriptionally regulated by NFATc2 in MDA-MB-231 [48]. As NFAT members (NFATc1-c4) are able to bind to the same DNA sequence [28], we hypothesized that NFATc1 could also bind to GPC6 promoter regions. The results of our ChIP assay confirmed that in MDA-MB-231, GPC6 is a transcriptional target of NFATc1, which is downstream of TRPC3.

Long-term knockdown of TRPC3 led to a decreased expression of GPC6, which is known to be a positive regulator of MDA-MB-231 cell migration [48]. Cell migration is a complex process with multiple steps involving the assembly and disassembly of FAs [36]. Regular FA dynamics ensures the efficiency of cell migration [36]. Cytoskeleton organization and FA formation are initial processes for maintaining a high cell migration rate in cancer cells [36, 42]. A decrease in disassembly would increase the size of FAs and decrease the cell migration [36, 42]. On the other hand, actin-binding vinculin is commonly used to represent the peripheral FAs [9]. Intracellular calcium signaling plays a prominent role in modulating FAs turnover to maintain an optimal migration [40]. Previous studies showed that in breast cancer cells, blocking calcium increased peripheral actin bundles and impaired FAs turnover as revealed by the increased size of the FAs [47]. For instance, TRPM7 was found to positively regulate cellular tension, modify FA number and change directed cell movement in breast cancer cells [27]. Our findings interestingly revealed that long-term knockdown of TRPC3 induced larger, elongated peripheral actin-bound FAs in MDA-MB-231 and led to a decrease of migration of TNBC cells. In our study, we employed both wound healing assays and single-cell immunostaining to investigate the effect of TRPC3 on both collective and individual migration contexts. We acknowledge that collective migration, as observed in the wound healing assay, involves directionality and spatially polarized adhesions, which are not necessarily replicated in single-cell migration [32]. When a cell has limited innate directionality, its movement is more random. However, if a motogenic stimulus is presented as an external gradient or includes another type of guidance cue, the cell's steering or directional mechanism engages with its motility system to respond to the asymmetric external factor, resulting in directed migration [32]. Although the mechanics of collective migration differ significantly from those of single-cell movements [21, 32], focal adhesions are crucial for single cells to interact with the extracellular matrix, which forms the basis for collective cell migration [12]. Thüroff et al*.* have revealed that cytoskeletal forces and the spatial organization of cells significantly impact cell movement at both the single-cell and collective migration levels [39]. In our study, we aimed to elucidate the migration abilities of cancer cells by using wound healing assays and immunostaining of focal adhesions to assess the effect of TRPC3 knockdown on cell migration in both collective and single-cell contexts. Our results indicate that the migration ability of the cells was decreased in both scenarios. In the future, if the complex collective migration behavior has to be decoded at a higher resolution at single cell level, higher resolution imaging say using live-cell labelling and artificial intelligence approaches can be done [7, 15].

Heparan sulfate proteoglycans (HSPGs), a family of glycoproteins, have profound roles in a variety of cellular processes [30]. According to the subcellular localization, membrane HSPGs are classified into two groups, namely syndecans and glycosylphosphatidylinositol-anchored proteoglycans [glypicans (GPC)]. GPCs have six members (GPC1-GPC6) in mammalian cells. Emerging role of upregulation of GPCs in various cancers attracted our attentions [25]. Several studies have revealed that GPCs regulate cancer cell metastasis through modulating Wnt signaling as well as MAPK pathway [49]. In addition, interaction between HSPGs and FAs was known to regulate cell migration [4, 17]. As GPC6 was down-regulated in MDA-MB-231-shTRPC3 cells, we inferred that GPC6 is the intermediate player in modulating MDA-MB-231 cell migration. Vinculin plays a key role in FA formation and cell migration. Vinculin can stay in the active form by interacting with binding partners. In migrating cells, newly formed FAs are small dot-like, then mature into streak-shaped FAs. The formation and disassembly of FAs needs to be coordinated for controlling cell motility [9]. A previous study showed that when MDA-MB-231 cells were treated with the Ca^2+^ influx inhibitor SKF96365, large FAs were induced and cell migration was inhibited. The increased FA size was reported to be caused by the defective FA turnover [50]. Our results showed that GPC6 directly interacted with vinculin in MDA-MB-231, an exciting finding that has never been demonstrated. Interestingly, long-term knockdown of GPC6 inhibited MDA-MB-231 cell migration and changed vinculin staining patterns with larger FAs observed in cell periphery. The active streak-like vinculin present at the periphery of MDA-MB-231-shTRPC3 and MDA-MB-231-shGPC6 was classified as a major component of matured FAs. Given we found that 1) GPC6 was down-regulated in MDA-MB-231-shTRPC3 cells, 2) downregulation of both TRPC3 and GPC6 led to a decrease in cell migration, 3) GPC6 physically interacted with vinculin, a FA protein, and 4) downregulation of GPC6 altered the FA dynamics such that larger and longer FAs were found at the cell periphery, and that it is well-established that altered FA dynamics affect cell migration, these larger, stabilized actin-bound FAs indicated that long-term knockdown of GPC6 and TRPC3 changed the dynamics of FAs turnover, which in turn, impaired directed cell migration [19, 26].

## Conclusion

In conclusion, we demonstrated that in TNBC cells, Ca^2+^ influx through TRPC3 channel positively regulates NFATc1 translocation and GPC6 expression, determines the dynamics of FA turnover and positively regulates cell migration. This novel TRPC3-NFATc1-GPC6-vinculin signaling cascade was revealed to be an important determinant of the migration of TNBC cells.

## Supplementary Information

Below is the link to the electronic supplementary material.

Supplementary file1 (DOCX 3.07 MB)

## Data Availability

The datasets used and/or analyzed during the current study are available from the corresponding author on reasonable request.

## Abbreviations

*TRPC3*: Canonical transient receptor potential isoform 3
*NFAT*: Nuclear factor of activated T cells
*TNBC*: Triple-negative breast cancer
*GPC6*: Glypican 6
*FA*: Focal adhesion
*ChIP*: Chromatin immunoprecipitation
*Co-IP*: Co-immunoprecipitation
