# Supplementary material for: TRPC3-mediated NFATc1 calcium signaling promotes triple negative breast cancer migration through regulating glypican-6 and focal adhesion
Source: Pflugers Arch. 2024 Oct 22;477(2):253–72. doi: 10.1007/s00424-024-03030-y (PMC11762004; doi:10.1007/s00424-024-03030-y)
Supplement: Supplementary file 1 — Supplementary file1 (DOCX 3.07 MB) [file 424_2024_3030_MOESM1_ESM.docx]

**FIGURE 1**


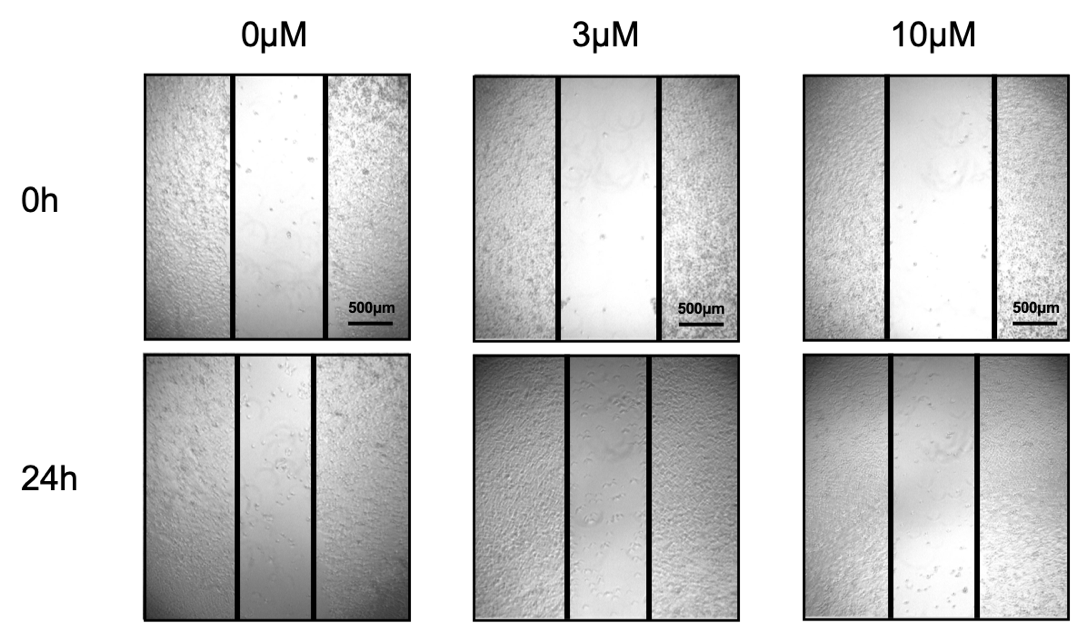
**A**

**
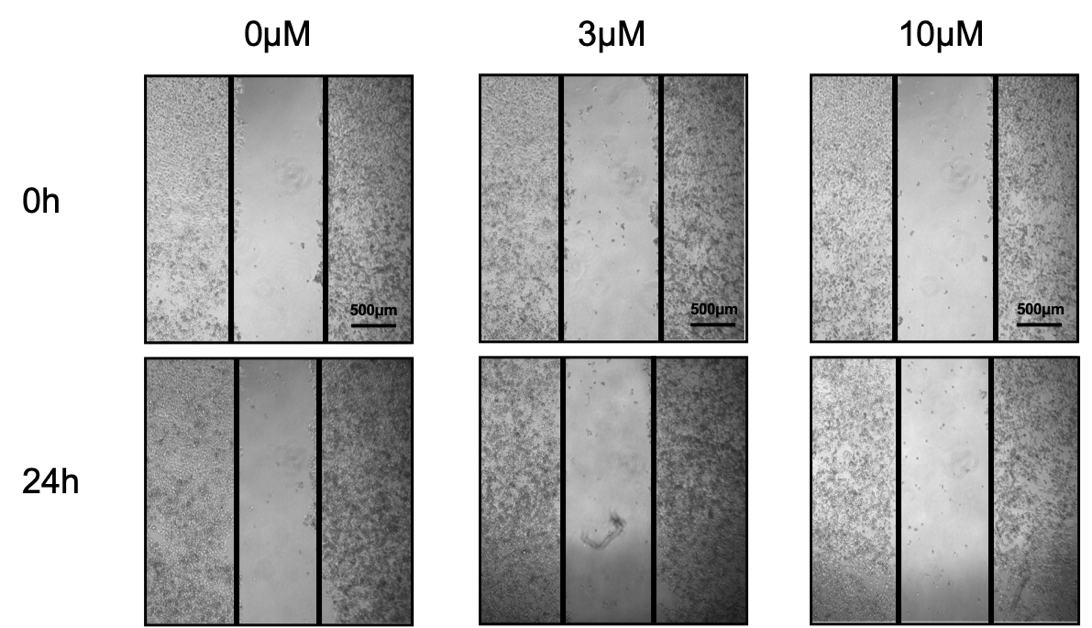
B**

**
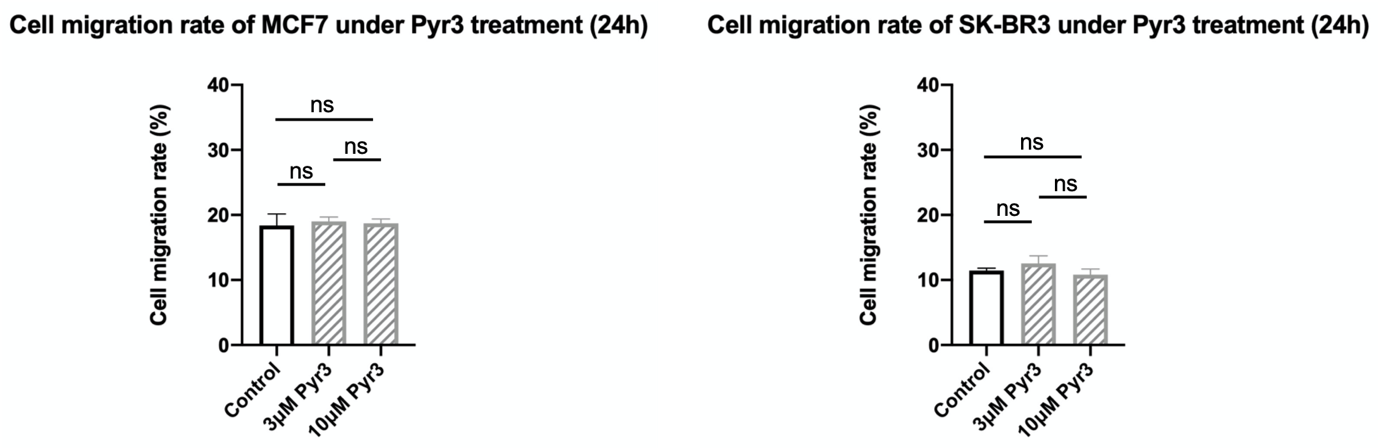
C**

**Supplementary Figure 1**

**Blockade of TRPC3 did not decrease migration in MCF-7 and SK-BR3.**

(A-B) Representative images showing the wound gap of MCF-7 and SK-BR3 at 0h and 24h after wound scratch in the wound healing assay under treatments with different concentrations of Pyr3, the pharmacological blocker of TRPC3. Scale bar: 500 μm.

(C) Summarized data on the cell migration rates 24h after wound scratch. Cell migration rates in the 3μM and 10μM Pyr3 treated groups were similar to the solvent control group. Values are mean ± SEM (n = 3). n.s.: not significant.

**FIGURE 2**

**
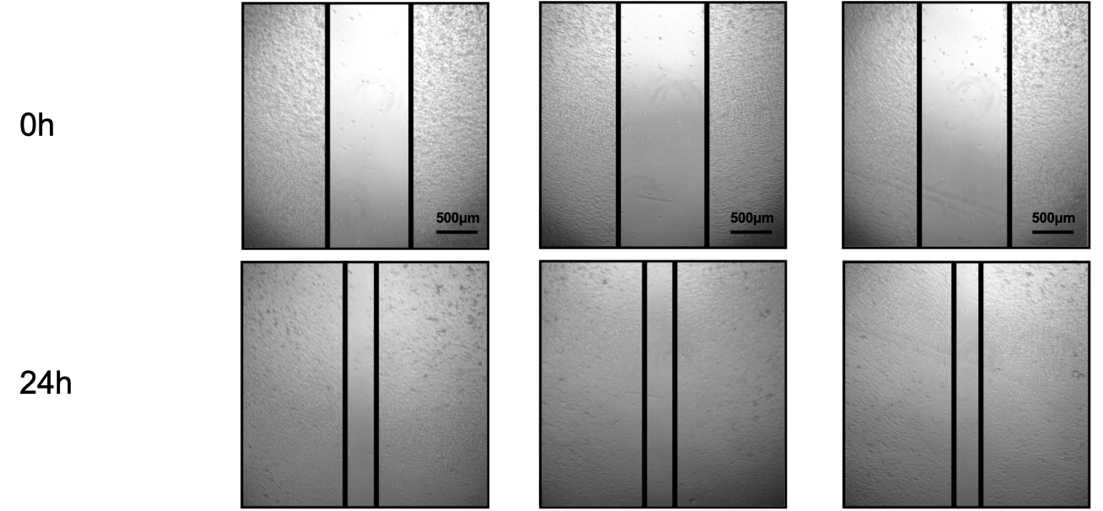
A**

**B**

**
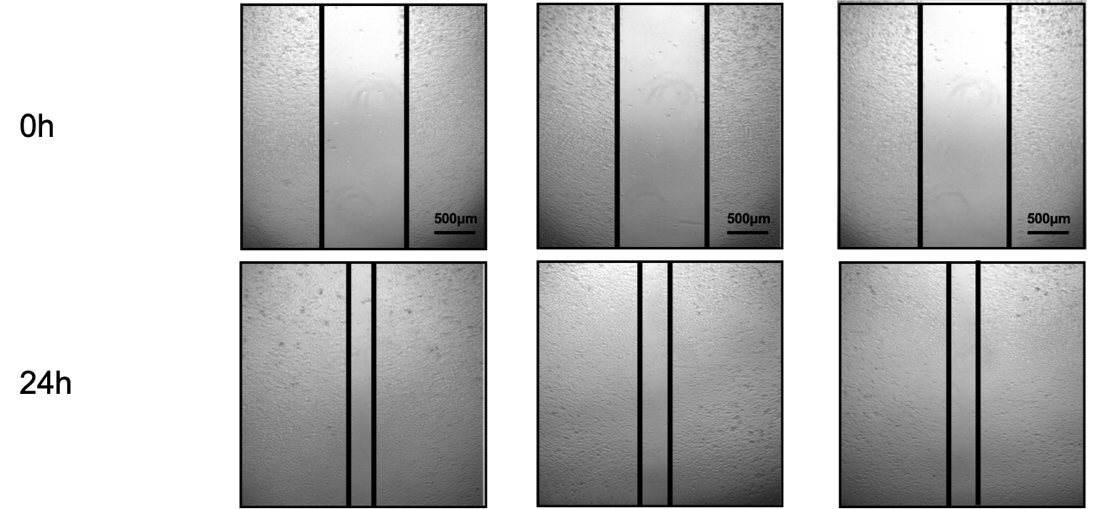
**

**
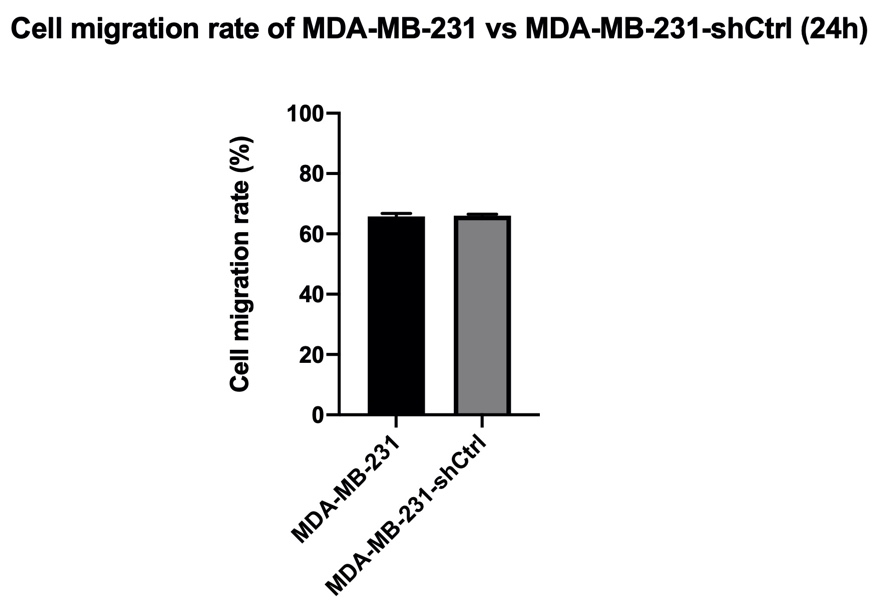
C**

**Supplementary Figure 2**

**Cell migration rates were similar in MDA-MB-231 and MDA-MB-231-shCtrl cells.**

(A-B) Representative images showing the wound gap at 0h and 24h after wound scratch in the wound healing assay in MDA-MB-231 and MDA-MB-231-shCtrl cells. Scale bar: 500 μm.

(C) Summarized data on the cell migration rates 24h after wound scratch. Values are mean ± SEM (n = 3). n.s.: not significant.

**FIGURE 3**

**
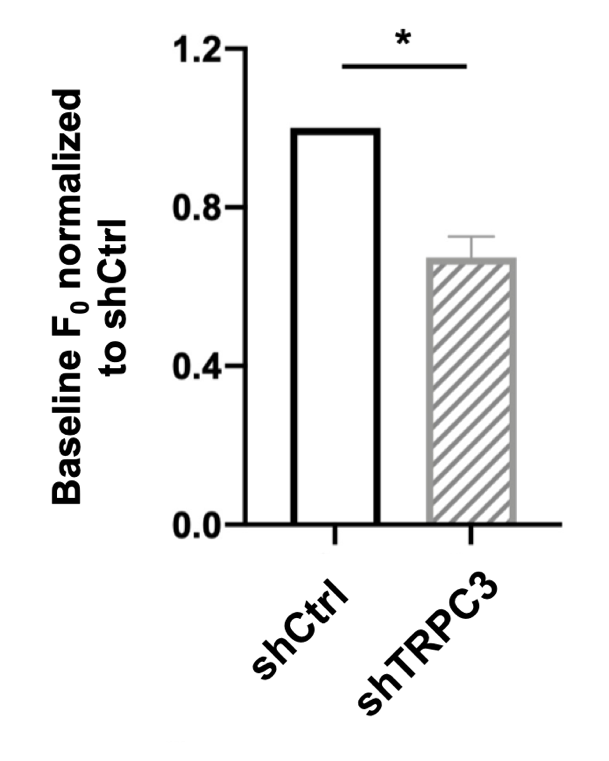
**

**Supplementary Figure 3**

**The basal Ca^2+^ level was significantly lower in the MDA-MB-231-shTRPC3 group when compared to the MDA-MB-231-shCtrl group.**

Baseline fluorescence value (F0) of both groups was normalized to the F0 of MDA-MB-231-shCtrl. MDA-MB-231-shTRPC3 cells were found to have a significantly lower level of basal Ca^2+^ . * P < 0.05.

**FIGURE 4**

**
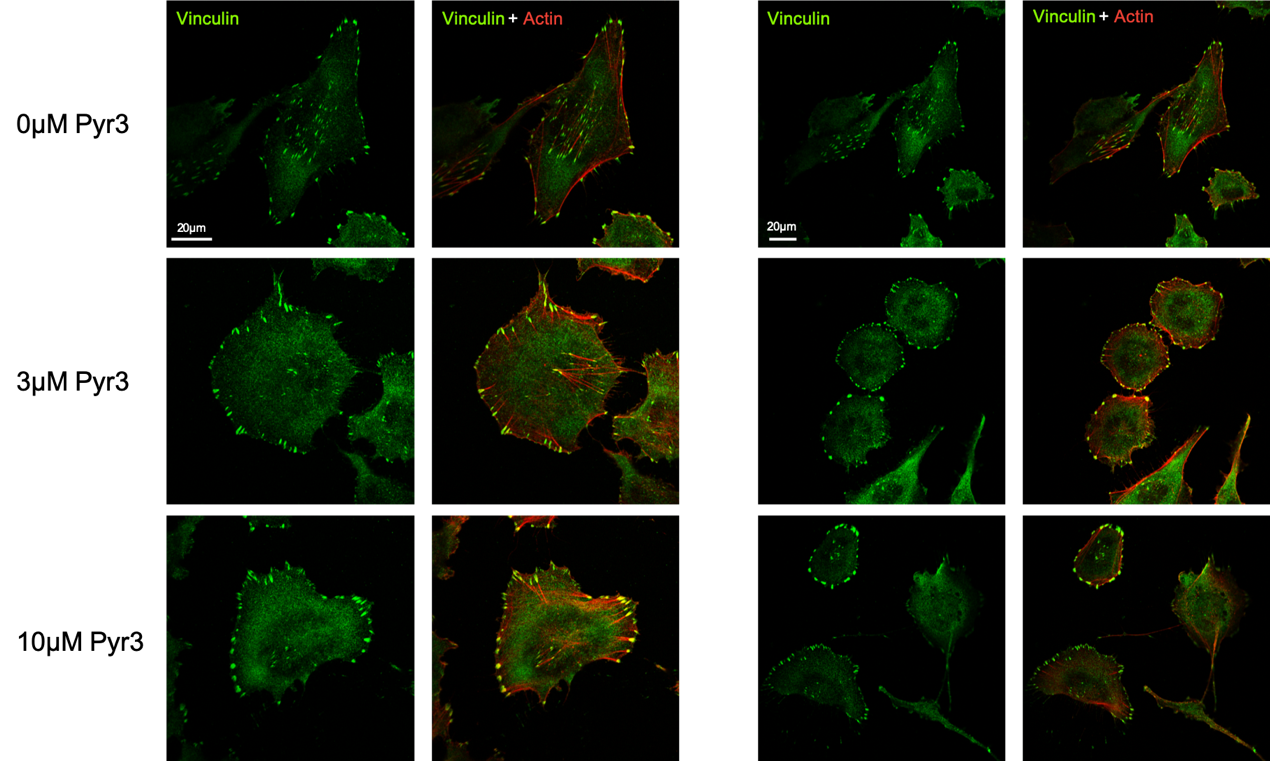
A**

**
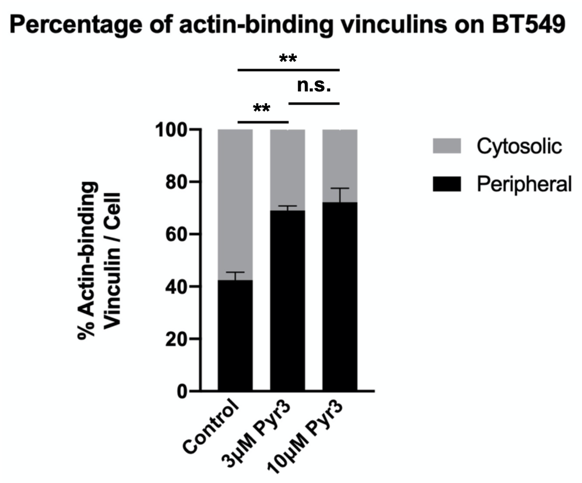
B**

**
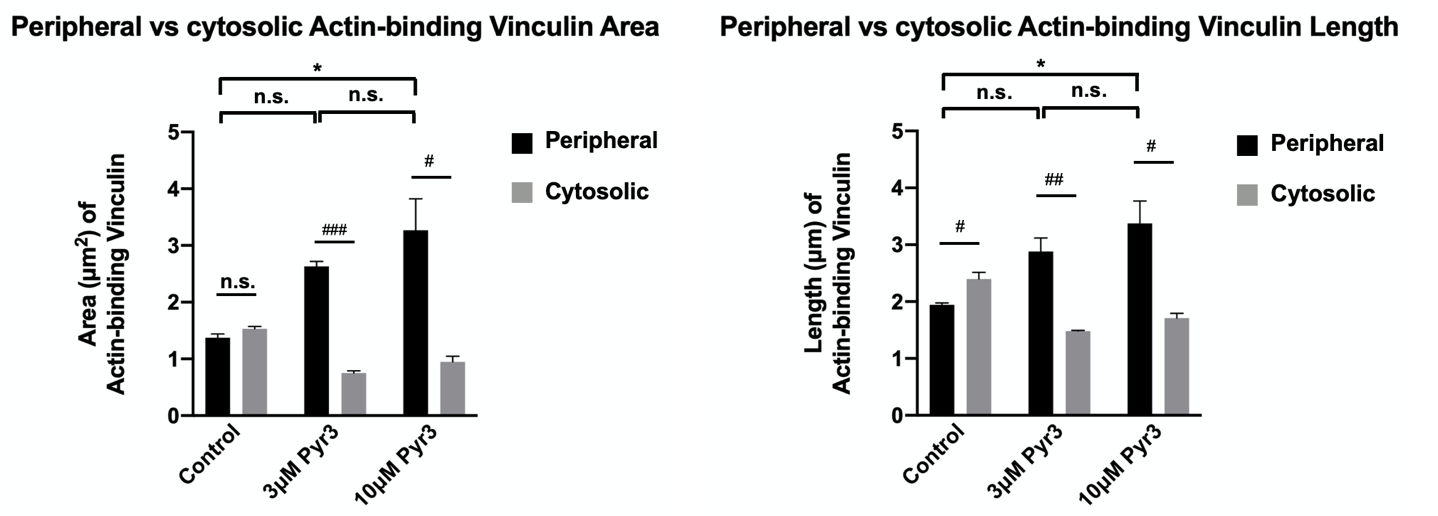
C**

**Supplementary Figure 4**

**Actin-binding vinculins distribution in BT-549 under Pyr3 treatment.**

1. Distribution pattern of actin-binding vinculins in BT-549. Cells were treated with 0μM, 3μM and 10μM Pyr3 for 24h before fixation. Fixed cells were stained with anti-vinculin (green) and phalloidin (to indicate actin; red). Scale bar: 20 μm.
2. Summarized data on the percentage of counts with actin-binding vinculin for those in the cytosol (grey bar) and in the cell periphery (black bar) to the total counts per cell. Blockade of TRPC3 increased the percentage of peripheral FAs in BT-549 cells.
3. Summarized data on the (left) area and (right) length of co-localized vinculin and actin. Both the area and the length of peripheral actin-binding vinculin (FAs) of the 10μM Pyr3-treated BT-549 were larger than the control cells. In addition, the area and the length of peripheral FAs were larger than those of cytosolic actin-binding vinculin in 3μM and 10μM Pyr3 treated cells while such was not observed in the solvent control group. The area and length of FAs were measured using the NIH Image J software. Values are mean ± SEM (n = 3). * P < 0.05 (among different experimental groups); # P < 0.05; ## P < 0.01; ### P < 0.001 (among peripheral and cytosolic data).
